# Comparative Genomic Analysis of Human Fungal Pathogens Causing Paracoccidioidomycosis

**DOI:** 10.1371/journal.pgen.1002345

**Published:** 2011-10-27

**Authors:** Christopher A. Desjardins, Mia D. Champion, Jason W. Holder, Anna Muszewska, Jonathan Goldberg, Alexandre M. Bailão, Marcelo Macedo Brigido, Márcia Eliana da Silva Ferreira, Ana Maria Garcia, Marcin Grynberg, Sharvari Gujja, David I. Heiman, Matthew R. Henn, Chinnappa D. Kodira, Henry León-Narváez, Larissa V. G. Longo, Li-Jun Ma, Iran Malavazi, Alisson L. Matsuo, Flavia V. Morais, Maristela Pereira, Sabrina Rodríguez-Brito, Sharadha Sakthikumar, Silvia M. Salem-Izacc, Sean M. Sykes, Marcus Melo Teixeira, Milene C. Vallejo, Maria Emília Machado Telles Walter, Chandri Yandava, Sarah Young, Qiandong Zeng, Jeremy Zucker, Maria Sueli Felipe, Gustavo H. Goldman, Brian J. Haas, Juan G. McEwen, Gustavo Nino-Vega, Rosana Puccia, Gioconda San-Blas, Celia Maria de Almeida Soares, Bruce W. Birren, Christina A. Cuomo

**Affiliations:** 1 Broad Institute of MIT and Harvard, Cambridge, Massachusetts, United States of America; 2 Department of Biology, Massachusetts Institute of Technology, Cambridge, Massachusetts, United States of America; 3 Institute of Biochemistry and Biophysics, Polish Academy of Sciences, Warszawa, Poland; 4 Laboratório de Biologia Molecular, Instituto de Ciências Biológicas, Universidade Federal de Goiás, Goiânia, Brazil; 5 Instituto de Ciências Biológicas, Universidade de Brasília, Brasília, Brazil; 6 Faculdade de Ciências Farmacêuticas de Ribeirão Preto Universidade de São Paulo, Ribeirão Preto, Brazil; 7 Unidad de Biología Celular y Molecular, Corporación para Investigaciones Biológicas, Medellín, Colombia; 8 Centro de Microbiología y Biología Celular, Instituto Venezolano de Investigaciones Científicas, Caracas, Venezuela; 9 Departamento de Microbiologia, Imunologia, e Parasitologia, Escola Paulista de Medicina, Universidade Federal de São Paulo, São Paulo, Brazil; 10 Instituto de Pesquisa y Desenvolvimento, Universidade do Vale do Paraíba, São José dos Campos, Brazil; 11 Instituto de Ciências Exatas, Universidade de Brasília, Brasília, Brazil; 12 Laboratório Nacional de Ciência e Tecnologia do Bioetanol – CTBE, São Paulo, Brazil; 13 Facultad de Medicina, Universidad de Antioquia, Medellín, Colombia; Progentech, United States of America

## Abstract

*Paracoccidioides* is a fungal pathogen and the cause of paracoccidioidomycosis, a health-threatening human systemic mycosis endemic to Latin America. Infection by *Paracoccidioides*, a dimorphic fungus in the order Onygenales, is coupled with a thermally regulated transition from a soil-dwelling filamentous form to a yeast-like pathogenic form. To better understand the genetic basis of growth and pathogenicity in *Paracoccidioides*, we sequenced the genomes of two strains of *Paracoccidioides brasiliensis* (Pb03 and Pb18) and one strain of *Paracoccidioides lutzii* (Pb01). These genomes range in size from 29.1 Mb to 32.9 Mb and encode 7,610 to 8,130 genes. To enable genetic studies, we mapped 94% of the *P. brasiliensis* Pb18 assembly onto five chromosomes. We characterized gene family content across Onygenales and related fungi, and within *Paracoccidioides* we found expansions of the fungal-specific kinase family FunK1. Additionally, the Onygenales have lost many genes involved in carbohydrate metabolism and fewer genes involved in protein metabolism, resulting in a higher ratio of proteases to carbohydrate active enzymes in the Onygenales than their relatives. To determine if gene content correlated with growth on different substrates, we screened the non-pathogenic onygenale *Uncinocarpus reesii*, which has orthologs for 91% of *Paracoccidioides* metabolic genes, for growth on 190 carbon sources. *U. reesii* showed growth on a limited range of carbohydrates, primarily basic plant sugars and cell wall components; this suggests that Onygenales, including dimorphic fungi, can degrade cellulosic plant material in the soil. In addition, *U. reesii* grew on gelatin and a wide range of dipeptides and amino acids, indicating a preference for proteinaceous growth substrates over carbohydrates, which may enable these fungi to also degrade animal biomass. These capabilities for degrading plant and animal substrates suggest a duality in lifestyle that could enable pathogenic species of Onygenales to transfer from soil to animal hosts.

## Author Summary

*Paracoccidioides* sp. are fungal pathogens that cause paracoccidioidomycosis in humans. They are part of a larger group of dimorphic fungi causing pulmonary infections in immunocompetent people, whereas many other fungi cause opportunistic infections. We sequenced the genomes of two strains of *Paracoccidioides brasiliensis* and one strain of the closely related species *Paracoccidioides lutzii*, and compared them to other fungal genomes. We found gene family expansions specific to *Paracoccidioides*, including the fungal-specific kinase family. By contrast we found that dimorphic fungi as a group have lost many genes involved in carbohydrate metabolism but retained most proteases. As the growth substrates for dimorphic fungi have not been well characterized, we tested a non-pathogenic relative, *Uncinocarpus reesii*, for growth on 190 carbon sources. We found that *U. reesii* is capable of growth on a limited set of carbohydrates, but grows more rapidly on a wide range of dipeptides and amino acids. Our analysis suggests that this genetic and phenotypic preference may underlie the ability of the dimorphic fungi to infect and grow on animal hosts.

## Introduction

*Paracoccidioides*, a dimorphic fungal pathogen, has infected approximately 10 million people in Latin America [1]. Each year, thousands of these infections develop into a systemic mycosis termed paracoccidioidomycosis, which requires prolonged treatment and has a high rate of relapse and complications [2], [3]. Despite the prevalence of *Paracoccidioides* infection, there is no estimate of the disease burden measured in disability-adjusted life years [4]. Other dimorphic fungi from the genera *Coccidioides*, *Blastomyces*, and *Histoplasma* cause over one million new infections each year in the United States alone [5], making dimorphic fungi the most common etiological agents of fungal pulmonary infection of otherwise healthy hosts [1], [6], [7]. All of these dimorphic fungi are pathogenic and during infection undergo a thermally regulated shift characterized by a morphological change between mycelial and yeast phases of growth [8]. These fungi are phylogenetically related, belonging to phylum Ascomycota, order Onygenales [9]. Onygenales also contains a number of non-dimorphic fungi including pathogens such as some *Microsporum* species and non-pathogens such as *Uncinocarpus*.

A common attribute of all dimorphic pathogens is the distinct growth conditions associated with temperature dependent alterations in morphological state. Specifically, a non-virulent filamentous form consisting of long chained cells producing asexual spores is observed in soil or in culture at 23°C, and a budding yeast form (or in *Coccidioides*, a related spherule/endospore form) in the host pulmonary system or in culture at 37°C. In filamentous form, dimorphic fungi are thought to be saprophytic, although whether they primarily decay plant or animal matter has been debated [10]. Infection of a mammalian host typically occurs following disruption of the fungus residing in soil and subsequent inhalation of airborne microconidia, which transform into a parasitic yeast form in the pulmonary alveolar epithelium [6].

Recent studies have begun to identify genes required for pathogenicity in dimorphic fungi [11]. These include a hybrid histidine kinase (*DRK1*), which controls the temperature dependent mold to yeast transition [12]. The dimorphic transition is concurrent with relocalization and organization of membrane lipids [13], [14], global changes in cell wall composition including alterations in carbohydrate polymers, such as glucan structure and chitin content [15], [16], [17], and temperature and other growth condition dependent shifts in gene expression [18], [19]. Some genes induced during the dimorphic shift are required for virulence, including two adhesins (*BAD1* and *SOWgp*), a calcium-binding protein (*CBP1*), and a catalase peroxidase [20].

The *Paracoccidioides* genus is composed of four distinct phylogenetic lineages (PS2, PS3, S1, and Pb01-like), which vary in virulence, culture adaptation, and induce different host immune responses [21], [22]. The three strains we selected for sequencing, Pb01, Pb03, and Pb18, represent three of the *Paracoccidioides* phylogenetic lineages. Strain Pb18 is a member of Species 1 (S1), which is composed of 38 isolates distributed across Latin America [21]. The Pb03 strain belongs to phylogenetic species 2 (PS2), which is composed of one Venezuelan and five Brazilian isolates. The extensively studied clinical isolate Pb01 is phylogenetically distinct from other strains and has been recently defined as a new species, *Paracoccidioides lutzii* [23]. In addition to interspecific variation, *Paracoccidioides* strains have been shown to contain extensive intraspecific genetic variability [24], [25], [26]. Comparing the genomes of the three isolates selected therefore allows identification of the features common across a wide range of diversity. In addition, the differences between the three strains provide a genome-wide pattern of the variation between strains in this genus.

To further elucidate the genomic basis of growth and virulence in dimorphic fungi, here we describe high quality reference genomes for all three *Paracoccidioides* strains. Using a combination of computational approaches, we characterize gene family content and metabolic pathways across dimorphic and related fungi, particularly with respect to carbohydrate and protein metabolism. We then test experimentally if predicted metabolic pathways correlate with carbon utilization using phenotype microarrays [27]. Only a few studies have characterized carbon utilization phenotypes in filamentous fungi (e.g., [28], [29]), and none of these studies have been conducted on Onygenales. We therefore screen the non-pathogenic Onygenale *Uncinocarpus reesii* for growth on 190 compounds as the sole carbon source, to better understand the metabolic capabilities of dimorphic fungi in mycelial form.

Our analysis comparing the *Paracoccidioides* genomes with diverse relatives from the order Onygenales has also provided key insights into genomic attributes that have contributed to the divergence of the *Paracoccidioides* lineage from other dimorphic fungal species, as well as the genetic diversity which differentiates *P. brasiliensis* strains from the *P. lutzii* (Pb01-like) species. These insights include a catalogue of unique genes and metabolic pathways that are conserved with close dimorphic relatives. Our study provides a basis from which to identify the underlying molecular differences that determine the infectious potency of *Paracoccidioides* strains and give rise to the clinical profiles attributable to paracoccidioidomycosis.

## Results

### Genome characteristics

**Figure 1:**
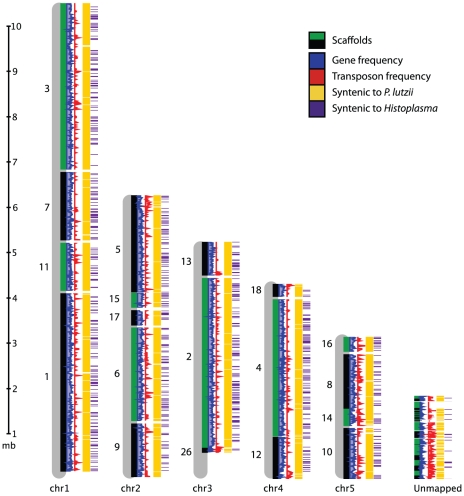
Genome organization of *P. brasiliensis*. Alignment of Pb18 supercontigs to the five chromosomes from the optical map are shown. Tracks (left to right) show gene frequency (blue), transposon frequency (red), synteny with *P. lutzii* (yellow), and synteny with *Histoplasma* (purple).

We produced 8-10X sequence coverage using Sanger technology for two strains of *P. brasiliensis* (Pb03 and Pb18), and one strain of the recently classified related species *P. lutzii* (Pb01) [23] (Table 1, Materials and Methods). The sequence was assembled using Arachne [30], and scaffolds representing the mitochondrial genome were separated out. To assess assembly accuracy and completeness, we generated an optical map of the Pb18 strain consisting of five linkage groups which likely correspond to complete chromosomes (Figure 1, Table S1). A total of 94% of the Pb18 assembly could be anchored to the optical map; the unanchored sequence was repetitive and similar in size to the unaligned regions of the optical map.

**Table 1:** Assembly and gene statistics.

	*P. lutzii*	*P. brasiliensis*	
		Pb03	Pb18
Coverage	8.0X	8.9X	9.8X
Assembly size (Mb)	32.9	29.1	30.0
Total contig length (Mb)	32.6	28.8	29.4
Scaffolds	111	65	57
Scaffold N50 (Mb)	1.02	1.97	2.13
Contigs	885	552	669
Contig N50 (kb)	84.3	114.9	109.7
Quality ≥Q40 (%)	98.9	98.9	98.9
GC (%)	42.8	44.5	44.4
Predicted protein-coding genes	9,132	7,875	8,741
Dubious genes	1,002	265	699
High-confidence genes	8,130	7,610	8,042
Mean gene length (nt)	1,814	1,833	1,802
Mean coding sequence length (nt)	1,330	1,433	1,346
Mean intron length (nt)	126	140	132
Mean intron number per gene	3.1	2.5	2.8
Mean exon number per gene	4.1	3.5	3.8
GC exonic (%)	49.8	50.4	50.1
GC intronic (%)	41.7	42.4	42.3
Mean intergenic length (nt)	1,799	1,848	1,628
tRNAs	118	103	103
Transmembrane proteins	1121	1057	1084
Secreted proteins	297	291	322
GPI-anchored proteins	61	63	62

The two *P. brasiliensis* genomes, Pb18 and Pb03, are similar in size (30.0 Mb and 29.1 Mb respectively) (Table 1). The *P. lutzii* genome is nearly 3 Mb larger at 32.9 Mb. The total number of initial predicted genes varies between 7,875 in Pb03 to 9,132 in *P. lutzii*. We assessed the evidence supporting each gene prediction in all three genomes, and flagged as dubious those with low levels of support in each of the three genomes. Once accounting for dubious genes, the set of high confidence genes is more similar across the three genomes, varying between 7,610 in Pb03 to 8,130 in *P. lutzii* (Table 1).

While all three *Paracoccidioides* genomes are highly syntenic, Pb18 and Pb03 share a significantly higher percentage of sequence similarity (∼96%) to each other in comparison to *P. lutzii* (∼90%) (Figure 1, Figure S1A, Table S2). Substantial synteny also exists between *Paracoccidioides* and *Histoplasma*, totaling 2.4 Mb; about half this amount is syntenic between *Paracoccidioides* and *Coccidioides* (Figure S1B, Table S2). Additionally, we used DAGchainer [31] to identify syntenic blocks of 6 or more genes between the species of *Paracoccidioides*. For *P. brasiliensis* strains Pb03 and Pb18, 95% and 93% of the genome was represented by syntenic blocks. For *P. lutzii*, 85% of the genome was represented by syntenic blocks. We then identified gene ontology (GO) terms enriched in these unique regions, by comparing all unique regions of all genomes to all non-unique regions of all genomes. While we mostly found transposable element-related PFAM and GO terms significantly over-represented (Table S3), some other PFAM domains were enriched in unique regions, such as protein kinases (PF00069, Fisher's exact test, p<2e-11) and histones (PF00125, Fisher's exact test, p<3e-7).

We identified single nucleotide polymorphisms (SNPs) between the strains by comparing the sequence reads for each strain to the Pb18 assembly. A total of 176,267 SNPs were found between the two *P. brasiliensis* strains, Pb18 and Pb03; normalized across high quality aligned positions, this represents a rate of 1 SNP every 132 bases. A higher number and rate of SNPs are found between *P. brasiliensis* (Pb18) and *P. lutzii*. A total of 501,313 SNPs were identified; across high quality aligned positions this represents a rate of 1 SNP every 26 bases. SNPs from each comparison are evenly distributed across the Pb18 genome (Figure S2). A small number of higher diversity regions are observed between Pb03 and Pb18, which include 67 genes. Relative to genes outside of these regions, the set of high diversity genes is enriched for PFAM domains RPEL (PF02755, involved in DNA binding) and PT (PF04886, a repeat domain) (Fisher's exact test, p<7e-4 for both domains). The high diversity genes are not enriched for any GO terms.

### Expansion of repetitive sequence in *P. lutzii*

The *Paracoccidioides* genomes contain all the basic transposable element types (Table 2). Transposons constitute approximately 8-9% of the *P. brasiliensis* Pb03 and Pb18 genomes and twice this amount in the *P. lutzii* genome (16%) (Table 2, Figure S3). On average the transposon sequences are 37-39% GC in the three genomes; this content is lower than the rest of the genome (43–45% GC) and the larger transposon territory in *P. lutzii* likely contributes to the lower overall GC content for this assembly (Table 1). Two major types of Class I elements (retrotransposons), LTR retrotransposons, and LINEs are present in the three genomes, however, no SINE elements were detected. There are more Ty3/Gypsy LTR retrotransposons than Ty1/Copia elements, which is typical for fungal genomes [32]. The number of LTR retrotransposons varies strikingly among the three genomes. The *P. lutzii* genome has a two-fold expansion of LTR elements compared to Pb18 and Pb03. By contrast, fewer LINE elements, predominantly Tad1 types, are found and their number is similar among these genomes (Figure S3).

**Table 2:** Repetitive element composition.

type	*P. lutzii*	*P. brasiliensis* (Pb03)	*P. brasiliensis* (Pb18)
DNA	2	10	10
DNA/TcMar	19	15	19
DNA/TcMar-Ant1	1	2	2
DNA/TcMar-Fot1	284	387	417
DNA/TcMar-Mariner	3	3	2
DNA/TcMar-Tc1	1	0	0
LINE	5	8	8
LINE/Tad1	82	62	88
LTR	277	101	124
LTR/Copia	165	80	41
LTR/Gypsy	442	85	199
Total elements	1,281	753	910
Percent of assembly	15.9%	7.7%	9.2%

Most types of DNA transposons were identified in each of the three genomes, but many are found in fewer copies in *P. lutzii*. The Class II repertoire in *Paracoccidioides* includes Mariner, hAT, Fot and Ant1 families. The predominant Fot1 class is more prevalent in the two *P. brasiliensis* genomes, Pb03 and Pb18, a possible sign of recent activity. Tc1/Mariner elements are reported to contain eight different subclasses and are also more prevalent in Pb03 and Pb18 [33].

*P. lutzii* has a new Tc1 type element which is absent in the Pb18 and Pb03. The DNA sequence is distinct from all sequences present in GenBank, but contains a protein with sequence similarity to the Impala *Fusarium* element, an *Aspergillus niger* sequence (GI:145235099), and an *Aspergillus oryzae* sequence (GI:169772711). These elements each encode a transposase classified as a Transposase_5 (PF01498). The Transposase_5 domain is found in three subtypes within the *Paracoccidioides* genomes, with one type universal to all strains, the second present only in *P. lutzii* and Pb18 but in different ratios, and the third type specific to *P. lutzii* (Figure S4).

Low complexity repeats are also expanded in *P. lutzii* relative to Pb03 and Pb18. *P. lutzii* contains about 50% more AT-rich low complexity repeats (Table S4). The three genomes contain similar amounts of simple repeats. This expansion of low complexity repeats and of transposable elements in *P. lutzii* account for the majority of the 3.0 Mb increased genome size. While genes specific to *P. lutzii* are not enriched for any GO terms, they are enriched for zinc finger (PF00098) and histone (PF00125) PFAM domains (Fisher's exact test, p<0.05).

### Mitochondrial genome variation

The mitochondrial genome assembly for Pb03 totals 75 kb, similar to that published for Pb18 [34]. The *P. lutzii* assembly only covers about half of the mitochondrial genome, a total of 31 kb (Table S5). The mitochondrial genomes of Pb03 and Pb18 are co-linear and share 90.0% identity across 87.0% of Pb03. Regions aligning between *P. lutzii* and Pb18 are also highly similar, sharing 89.6% identity, but cover only 54.6% of *P. lutzii*; the lower fraction of the mitochondrial genomes that align across these genomes further highlights the divergence of the two species.

While all three mitochondrial genomes contain similar gene content, there are differences in splicing and gene order. The NADH dehydrogenase subunit 1 (*nad1*) is spliced in the Pb18 genome [34] and Pb03, but is unspliced in *P. lutzii*. *Nad1* is also unspliced in the mitochondrial genomes of other Onygenales, including *Histoplasma capsulatum* and *Trichophyton rubrum* [35], suggesting that the *nad1* intron was acquired in the *P. brasiliensis* lineage after divergence from *P. lutzii*. Another difference is in the *apocytochrome b* (*cob*) gene; in *P. lutzii* there is a 376 base insertion in exon 2 of this gene [36]. There is also a difference in splicing of the *cox1* gene between the two *P. brasiliensis* genomes. Pb18 contains an intron between exons 5 and 6 that is not found in Pb03; this region in Pb18 contains a group I intron RFAM motif and regions homologous to the LAGLIDADG endonuclease. One difference in gene order is also observed. Two maturase proteins are found in *P. brasiliensis* Pb18, within introns in the *cox1* and *cob* genes [34]. In the *P. lutzii* assembly, there is a maturase protein in a different location than in Pb03 and Pb18, between the *atp9* and *cox2* gene. These differences further establish the extensive interstrain genetic variation in *Paracoccidioides*.

### Gene family evolution

**Figure 2:**
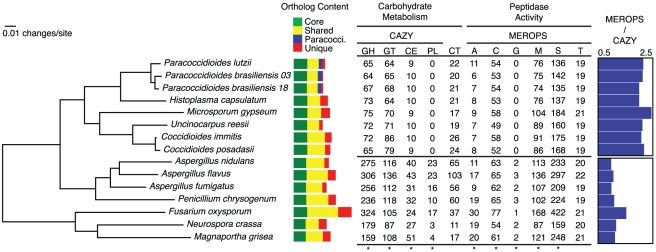
Phylogeny of the dimorphic fungi, shared gene content, and clade-specific carbohydrate and protein metabolism. The phylogeny was inferred by maximum likelihood using RAxML [103] from a concatenated alignment of 309 single copy orthologs shared across all taxa. All nodes on the phylogeny were supported with bootstrap values of 100%. Colored bars indicate orthologs shared across all taxa (green), orthologs shared across some taxa (yellow), *Paracoccidioides*-specific (blue) and species-specific genes (red). Numbers of genes for each taxon involved in carbohydrate metabolism and peptidase activity are shown. Categories with asterisks at the bottom of the column are significantly enriched in the non-dimorphic fungi relative to the dimorphic fungi. Carbohydrate active enzyme (CAZY) categories include glycoside hydrolases (GH), glycosyltransferases (GL), carbohydrate esterases (CE), and pectate lysases (PL). Numbers of carbohydrate transporters (CT) are also listed. Peptidase (MEROPS) categories include aspartic (A), cysteine (C), glutamic (G), metallo (M), serine (S) and threonine (T). The ratio of MEROPS genes to CAZY genes for each genome is shown on the right.

In order to study gene family evolution in the dimorphic fungi, and in the *Paracoccidioides* lineage in particular, we identified 20,713 orthologous gene clusters across 15 fungal genomes, including 8 from Onygenales and 7 outgroups from the more inclusive clade Pezizomycotina (the filamentous Ascomycetes). A phylogenetic tree built using 309 single-copy orthologs from this analysis showed universally strong support for previously hypothesized phylogenetic relationships both between the dimorphic fungi and other Onygenales, and between the Onygenales and other Ascomycetes (Figure 2, [10], [37]). Two major lineages exist within Onygenales, one containing the dimorphic pathogens *Paracoccidioides* and *Histoplasma*, and the other containing the dimorphic pathogen *Coccidioides* nested within non-dimorphic Onygenales including *Microsporum* and *Uncinocarpus*.

The phylogenetic distribution of these ortholog clusters highlights the conserved and species specific fractions of the *Paracoccidioides* genomes (Figure 2); 995 clusters were specific to *Paracoccidioides*, and an additional 195 were specific to *Histoplasma* and at least one strain of *Paracoccidioides*. Genes in these sets had few functional predictions – only 8% of *Paracoccidioides*-specific genes and 21% of *Histoplasma* and *Paracoccidioides* -specific genes had predicted GO functions. There were 33 clusters unique to and present in all dimorphic fungi, the largest of which was a protein kinase in the recently described fungal-specific kinase family FunK1 [38]. No clusters were specific to all animal pathogens in the phylogeny.

To analyze functional gains and losses we mapped gene ontology (GO) annotations to all 15 genomes using Blast2GO [39]. We then used Fisher's exact test to identify significantly over- or under-represented GO terms between groups of interest: dimorphic fungi versus related non-dimorphic fungi, *Paracoccidioides* and *Histoplasma* versus *Coccidioides*, *Uncinocarpus*, and *Microsporum*, and *Paracoccidioides* versus *Histoplasma* (Table S6). A small number of GO terms were significantly over- or under-represented in the *Paracoccidioides* and *Histoplasma* versus *Coccidioides*, *Uncinocarpus*, and *Microsporum* comparison (Table S6). Further examination of GO terms enriched in the *Coccidioides* clade revealed these fungi encode a larger repertoire of metallo and serine peptidases than the *Paracoccidioides* clade. The *Paracoccidioides* clade was enriched for zinc-ion/DNA binding proteins, although this primarily resulted from a highly reduced number of zinc-ion/DNA binding proteins in *Microsporum*. No GO terms were significantly enriched in the *Paracoccidioides* versus *Histoplasma* analysis, suggesting the differences between these two clades do not include major differences in known functional categories; however, differences may exist for genes with currently unknown functions.

### Carbohydrate metabolism

Relative to other fungi, Onygenales, including the dimorphic fungi, showed a significantly reduced gene content with respect to many carbohydrate metabolism and transport related GO terms (Table S6). To further examine this reduction, we identified and compared all carbohydrate active enzymes (CAZY) for the genomes in our phylogenetic analysis [40], and a summary of the results are shown in Figure 2 (family-specific classification shown in Table S7). Onygenales have significantly fewer glycoside hydrolases, glycosyltransferases, carbohydrate esterases, and polysaccharide lyases than their non-dimorphic relatives (p = 0.008 for all comparisons, Mann-Whitney U test, Bonferroni corrected), although they do not have significantly different numbers of carbohydrate transporters. Ancestral character state reconstruction using GeneTRACE [41] found that no CAZY ortholog clusters were gained at the root of the Onygenales, while 142 were lost. This suggests that carbohydrate metabolism was reduced in Onygenales rather than expanded in their relatives.

Reductions in plant degradative genes have been previously noted in dimorphic fungi, and it has been hypothesized that dimorphic fungi consume decaying animal matter rather than plant matter while living in the soil [10]. However, *Paracoccidioides* in particular has been reported to grow on media with cellulose or xylan as the sole carbon source suggestive of a metabolism supporting plant decay [42]. Furthermore, a genomic signature such as the number of carbohydrate-degradation genes is not necessarily an indicator of ability to digest carbohydrates, as *Trichoderma reesei*, the main industrial fungal source of cellulases, encodes one of the smallest repertoires of cellulase genes in all fungi [43].

To investigate the catabolic capabilities of *Paracoccidioides* and other Onygenales, we characterized the metabolic pathways of all genomes in our phylogenetic analysis using Pathway Tools [44]. Our analysis indicated that the *Paracoccidioides* genome encodes for the catabolic pathways necessary to break down plant cell wall derived monosaccharides D-glucose, D-xylose, and D-mannose, and D-galactose, but not L-arabinose, suggesting despite an overall reduction in carbohydrate metabolism genes they are capable of degrading the most abundant plant sugars found in cell walls. Based on the presence of these pathways and previous growth experiments, we hypothesize that Onygenales, including the dimorphic fungi, are capable of feeding on cellulosic plant material components while growing in the soil.

### Protein catabolism

Degradation of proteins can support growth and provide an alternative carbon source to carbohydrates, and also is important for fungal pathogenesis [45]. We therefore identified and classified all peptidases in the 15 genomes using the MEROPS classification system [46], and summarized the results in Figure 2 (family-specific classification is shown in Table S8). The Onygenales had significantly fewer aspartic, cysteine, glutamic, metallo, and serine proteases than the non-dimorphic fungi (p<0.05 for all comparisons, Mann-Whitney U test, Bonferroni corrected). In particular the Onygenales completely lack the glutamic proteases normally common to filamentous fungi [47], and which have been shown to be essential for hyphal growth in the fungus *Talaromyces emersonii* [48]. Threonine proteases are conserved between the Onygenales and related fungi and the total numbers were not significantly different. Ancestral character state reconstruction of proteases showed an overall loss of proteases at the root of the Onygenales; 64 ortholog clusters were lost while only one, an M35 fungal metalloendopeptidase, was gained. However, while the Onygenales appear to have a smaller overall repertoire of proteases than their relatives, the ratio of proteases to carbohydrate active enzymes is much higher in the Onygenales (Figure 2), suggesting proteins are an important source of food for this order of fungi. Furthermore, based on metabolic pathway reconstruction, the Onygenales appear to retain all amino acid degradation pathways present in their relatives (http://fungicyc.broadinstitute.org).

Some peptidase families, namely S8 (serine endopeptidase subtilisins) and M35 (fungal metalloendopeptidases), were previously reported to be expanded in *Coccidioides* relative to other fungi [10]. The S8 and M35 families are not similarly expanded in *Paracoccidioides* nor in *Histoplasma*; S8 proteases are present in large numbers in *Fusarium oxysporum* and *Magnaporthe grisea*, and the M35 in *Aspergillus flavus* and *Magnaporthe grisea*, respectively (Table S8). Therefore these protease expansions are not general features of the dimorphic Onygenales. No individual families were expanded only in *Paracoccidioides* or in both *Paracoccidioides* and *Histoplasma* relative to the other fungi.

We compared the *Paracoccidioides* peptidase repertoire to proteins predicted to be secreted by SIGNALP [49], and identified 30–34 secreted proteases in each genome, including representatives of each catalytic type found in the complete set of *Paracoccidioides* peptidases. *Paracoccidioides* were predicted to have 30 to 35 secreted proteases, approximately half as many as in *Coccidioides*, which contain 62 to 63. While there were no secreted peptidases uniquely shared in *Paracoccidioides* and *Histoplasma* compared to the remaining dimorphic fungi, *Paracoccidioides* had a uniquely secreted ubiquitinyl hydrolase (family C19) and ATP-dependent metalloendopeptidase (family M41). As suggested by a previous survey based on ESTs [11], the *Paracoccidioides* genomes do not contain a homolog of the secreted protease Mep1, an M35 family member and important virulence factor in *Coccidioides* which helps the fungus evade detection by the host immune system [50]. Additionally, we identified a Raf-1-kinase-inhibitor-like protein secreted by all *Paracoccidioides* species. Raf-1 plays a critical role in cell differentiation [51], including that of immune cells. It is possible that this inhibitor plays a role in protecting *Paracoccidioides* from macrophages.

### Carbon utilization in soil relative *U. reesii*

**Figure 3:**
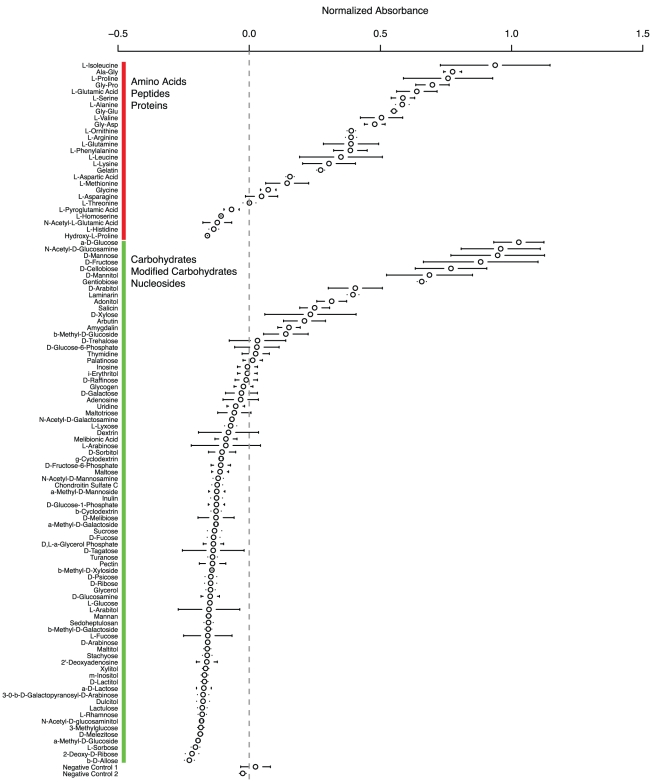
Growth by *Uncinocarpus reesii* on carbohydrates, amino acids, and peptides as the sole carbon source after a 7-day period. The experiment was done in triplicate; circles represent the normalized median absorbance at 590 nm and bars represent standard error. The dotted line indicates 0 normalized absorbance. Numerical growth values for all compounds can be found in Table S9.

As the ratio of proteases to carbohydrate active enzymes is much higher in the Onygenales than their relatives (Figure 2), we hypothesize that proteins may be an important source of food for the Onygenales relative to carbohydrates. In order to test this hypothesis, we utilized phenotype microarrays [27], which measure an organism's ability to grow on carbohydrates, amino acids, carboxylic acids, and a number of other compounds as the sole carbon source. Only a small number of these screens have been conducted on filamentous fungi (e.g., [28], [29]), and none of these studies examined Onygenales. As a first analysis for this group of fungi, we targeted a non-pathogenic model with similar gene content to *Paracoccidioides*. *Uncinocarpus reesii*, a non-pathogenic soil dwelling fungus in the Onygenales, is the closest non-pathogenic relative of *Paracoccidioides* for which a complete genome sequence is available. Metabolic pathway reconstruction for the four genera of Onygenales in our comparative analysis showed that of 33 examined amino acid and carbohydrate degradation pathways, *Paracoccidioides, Coccidioides*, *Histoplasma*, and *U. reesii* all shared identical conservation (presence or absence) for 27 pathways. (Table S9, http://fungicyc.broadinstitute.org). Only pathways for the degradation of D-galactose, meliobiose, asparagine and threonine were present in *Paracoccidioides* but not *U. reesii*, while a pathway for ornithine degradation was present in *U. reesii* but not *Paracoccidioides* (Table S9). Based on this high degree of phylogenetic relatedness and overlap of metabolic pathways among the Onygenales, we utilized *U. reesii* to better understand *Paracoccidioides* metabolism by growing *U. reesii* on 190 metabolic compounds, including 31 amino acids and peptides and 81 carbohydrates, over a seven day timecourse. Growth of *U. reesii* on all carbohydrates, peptides and amino acids at the seven day timepoint is shown in Figure 3 (complete data in Table S10).

*U. reesii* showed a limited ability to grow on carbohydrates, oligosaccharides, and carbohydrate-polymers (positive growth on 12 of 81 compounds tested) (Figure 3, Table S10), which was largely congruent with the degradation pathways predicted from the genome (Table S9). *U. reesii* grew well on monosaccharides D-glucose, D-mannose, and D-xylose in at least two of the three replicates. This fungus also grew well on glucose oligosaccharides with β 1-3 linkage such as gentiobiose, and β 1-4 D-cellobiose, the principal component and linkage in the cellulose polymer. The algae produced polymer laminarin, that contains both β 1-3 and β 1-6 linked glucose, was also found to be a growth substrate for *U. reesii*. Based on our pathway analysis, *U. reesii* appears to lack the cellulosic sugar D-galactose degradation pathway; in accordance with this it did not grow on this substrate. Further, oligosaccharides containing D-glucose with α linkages, such as the animal glucose-polymer glycogen did not support growth by this species. Overall, *U. reesii* did not grow well on most types of carbohydrates but did grow well on several primary components of plant cell wall, suggesting that selective pressure on the Onygenales retained the metabolic pathway genes responsible for degrading components of cellulose and hemi-cellulose, despite global losses in carbohydrate metabolism genes.

In contrast to its limited growth on carbohydrates, *U. reesii* grew well on a relatively wide range of amino acids and peptides (positive growth on 19 of 31 compounds tested) (Figure 3, Table S10). *U. reesii* also grew well on gelatin, a hydrolytic product of collagen that is the major structural polymer of the extracellular matrix in animal epithelial tissue. These results differ substantially from those obtained in a similar experiment conducted on the filamentous ascomycete *Trichoderma reesei* [28]. In *T. reesei*, the 20 compounds tested in both studies which produced the highest growth were all carbohydrates, while in *U. reesii* 12 were carbohydrates while 8 were proteins (however, gelatin was not tested in the *T. reesei* study). *T. reesei* is utilized as an industrial production strain for the degradation of cellulosic biomass and therefore might be expected to prefer carbohydrates to proteins. By contrast, both the greater relative gene content of proteases to carbohydrate active enzymes and the robust ability to grow on a number of proteinaceous substrates in *U. reesii*, suggests that proteins in general may be a transitional food source for the Onygenales that may have allowed these species to degrade animal biomass and possibly establish pathogenic colonization through proteolysis coupled with the dimorphic transition.

### Fungi-specific protein kinases

**Figure 4:**
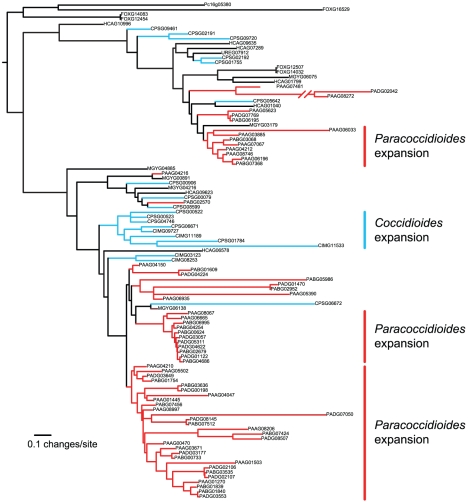
Phylogenetic relationships of FunK1 kinases estimated by maximum likelihood with RAxML [**103**]. Lineage-specific expansions can be seen in *Paracoccidioides* (red) and *Coccidioides* (blue).

Both *Paracoccidioides* and *Coccidioides* clades include large expansions of protein kinases. Detailed examination of these kinases revealed that they belong to the FunK1 family, recently described from the genome of the mushroom *Coprinopsis cinerea* [38]. Using an iteratively built hidden Markov model, we identified FunK1 genes in our 15 taxon data set of Pezizomycotina, including all examined Onygenales, *Fusarium*, and *Penicillium*. The FunK1 domain shares homology with 35% of the length of the well known Pkinase domain (PF00069). The shared region encompasses much of the C-terminal lobe of the domain, and contains regions that are essential for catalysis and are involved in substrate recognition and allosteric regulation [52]. By estimating the phylogeny of FunK1 genes, it is clear that the FunK1 genes have undergone independent lineage-specific expansions in both *Paracoccidioides* and *Coccidioides* (Figure 4). Further, as FunK1 proteins in *Paracoccidioides* were significantly enriched in non-syntenic regions versus syntenic regions (χ^2^ = 50.4, p<0.0001), this data supports the origin of FunK1 diversity by lineage-specific duplication events.

**Figure 5:**
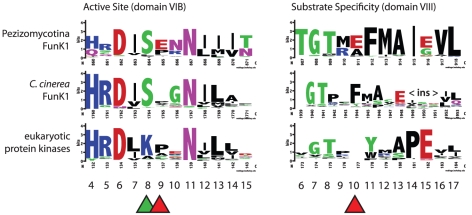
Conservation of FunK1 functional domains. The active site (domain VIB) and substrate specificity region (domain VIII) are shown for Pezizomycotina, including the dimorphic fungi (FunK1 Pezi), the mushroom *Coprinopsis cinerea* (Ccin), and ‘typical’ eukaryotic protein kinases (ePKs). The domain numbering follows standard protein kinase nomenclature [52]. FunK1 proteins share a unique serine at the active site position 8 (green triangle) compared to a lysine found in eukaryotic protein kinases. Additionally, FunK1 proteins have glutamate residues at positions VIB 9 and VIII 10 (red triangles), suggesting the recognition of arginine-containing substrates.

The active site and substrate specificity region vary for FunK1 proteins in Pezizomycotina, *C. cinerea*, and typical eukaryotic protein kinases (Figure 5). Many Pezizomycotina FunK1 subfamily members have glutamate residues at position 9 of the active site region (subdomain VIB), and position 10 of the substrate specificity region (subdomain VIII). Among eukaryotic protein kinases, protein kinase A (*PKA*) has glutamate residues at VIB10 and VIII10 that are involved in recognition of substrates with an arginine residue at the -3 position with respect to the phosphorylation site [53]. Modeling the Pezizomycotina FunK1 glutamate at position VIB9 indicates that it may fulfill the role of the *PKA* glutamate residue at position VIB10, suggesting that these kinases prefer similar substrates and, in this respect, differ from FunK1 family members from *C. cinerea*, which do not have glutamate residues at these positions.

To gain insight into the potential function of FunK1 genes, we examined published *Paracoccidioides* stage-specific expressed sequence tag (EST) databases for evidence of expression of these genes. Reads representing FunK1 genes were identified in EST libraries made from yeast-phase cells taken from infected mice [54]. However, only 5 FunK1 reads were identified out of 4,934 total reads, suggesting these genes are not highly expressed in infected hosts. Furthermore, none of these kinases were predicted to be secreted, suggesting their function is intracellular within the fungus. We also examined EST libraries made from mycelium and yeast cells *in vitro* [55], [56], and found 6 and 18 reads representing FunK1 genes, respectively. This suggests that FunK1 genes as a whole are not expressed narrowly in a specific stage, nor do they appear to be highly expressed in any stage examined. In total we detected expression of 11 of 21 FunK1 genes, suggesting that despite low levels of expression the majority of these genes are transcribed.

Histidine kinases (HK) sense host signals and trigger the transition from mold to yeast in both *Blastomyces dermatitidis* and *H. capsulatum* [12]. All sensor kinases in yeasts are hybrid HKs that contain both a histidine kinase domain and a receiver domain within a single polypeptide chain [57]. There are five sensor HK genes and a single histidine-containing phosphotransfer intermediate gene in all three *Paracoccidioides* genomes, and two genes encoding response regulators (Table S11). These genes are conserved as single copy orthologs in the Onygenales in our gene family analysis with the exception of one sensor kinase missing from *H. capsulatum* (Table S11). The conservation of these genes suggests that the HK regulatory pathways characterized in both *H. capsulatum* and *B. dermatitidis* may play a similar role in *Paracoccidioides*. HKs linked with two-component relays are absent in Metazoa, suggesting they could be used as potential targets for drug development in dimorphic fungi [12].

### Rapid evolution of dimorphism-related genes

To identify rapidly evolving genes which could be important for pathogenesis, we calculated the ratio of synonymous-to-nonsynonymous substitutions (dN/dS) within the *Paracoccidioides* and *Histoplasma* lineage, and examined enrichment for specific GO term categories. Proteins with zinc and DNA binding motifs, particularly transcription factors, represented the majority of rapidly evolving genes (Table S12). These include genes which play roles in dimorphism and cell morphogenesis in *Candida albicans*, including *RAP1* [58] and a C_2_H_2_-type conidiation transcription factor [59]. Additional rapidly evolving transcription factors are known to regulate morphogenesis and pathogenicity in other fungi, including an HMG box protein (*B. dermatitidis*, [60]), a homeobox domain protein (*Ustilago maydis*, [61]) and a cutinase palindrome-binding protein (*Fusarium solani*, [62]). Metal ion transporters represent another class of rapidly evolving genes. A large number of these are zinc transporters, and zinc acquisition from the environment has long been known to play a role in dimorphism in a number of fungi, including *H. capsulatum* [63] and *C. albicans* [64]. This rapid evolution of transcription factors in the *Paracoccidioides* and *Histoplasma* clade may represent continued adaptation of stage-specific gene expression and potentially of the regulation of dimorphism to signals and defenses from the host.

### Sexual reproduction in *Paracoccidioides*

The sequenced genomes provide evidence for a sexual stage in *P. brasiliensis*. For many years *Paracoccidioides* was considered an asexual and clonal microorganism (as reviewed in [9]), but recent data showing evidence for recombination have provided support for a sexual stage [21]. The presence of two mating type idiomorphs and other mating or meiosis specific genes suggest that an organism has a sexual stage, such as for the fungal pathogen *Aspergillus fumigatus* [65]. Each of the *Paracoccidioides* isolates sequenced contains one of the two mating type idiomorphs: α-box (*MAT1-1*) and HMG (*MAT1-2*). The *P. brasiliensis* Pb03 and Pb18 strains carry a *MAT1-2* idiomorph, while the *P. lutzii* strain carries a *MAT1-1* idiomorph [66], [67]. From the available ESTs, we identified two ESTs from a study with Pb18 [56] which best match the HMG-box protein PADG_06118; we also identified three ESTs from a study with the same strain of *P. lutzii* [55] that best match the alpha-box protein PAAG_05873. While this is a small number of EST sequences for each, this suggests that each mating type locus is actively expressed in these surveys. Additional representatives of *MAT1-1* or *MAT1-2* have been identified in different *Paracoccidioides* isolates [66], [67].

Conservation of genes necessary for mating and meiosis in the three *Paracoccidioides* genomes provides further support for a sexual stage. We identified orthologs of known regulators of the mating and meiotic process (Text S1 part 4, Tables S13, S14) from a wide range of fungi. Most genes involved in the mating process or mating signaling are highly conserved in *Paracoccidioides* and other dimorphic fungi; some components of the mating signaling signal transduction cascade are not conserved but their loss is not specific to *Paracoccidioides* (Table S13). Nearly all of a core set of genes involved in chromosome cohesion and recombination, which are conserved among sexual eukaryotes [68], are found in *Paracoccidioides*. One exception is that an ortholog of *HOP2* is missing in *Paracoccidioides* as well as *H. capsulatum* and *A. fumigatus*. In addition, many of the genes involved in sexual development, the cell cycle and meiosis, and sporulation are highly conserved in *Paracoccidioides* and other dimorphic fungi (Table S14). Expression of 78% of the mating and 72% of the meiosis genes can be detected from ESTs (Tables S13 and S14). This analysis suggests that all the genes required to support a sexual stage are present in the *Paracoccidioides* genomes, and that expression can be detected for most of these genes.

### Secondary metabolism

Secondary metabolites produced by *Paracoccidioides* may play important roles in virulence; potential secondary metabolite gene clusters are highly conserved among the three genomes. Three nonribosomal peptide synthetase (NRPS) and three polyketide synthase (PKS) genes were identified in each genome; an additional three genes have some similarity to NRPS and one to PKS (Table S15). Of this total group of ten, eight have adjacent genes which could form a secondary metabolite gene cluster (SMGC). The NRPS, PKS, and accessory genes are all conserved as orthologs in syntenic regions between the *Paracoccidioides* genomes. The three PKS encode typical fungal reducing PKS [69] and contain two domain structures. One of the three is missing an enol reductase (ER) domain, similar to other PKS of the lovastatin nonaketide group; this gene is conserved in all dimorphic fungi. The other two PKS genes contain an ER domain, and neither is highly conserved. One is specific to *Paracoccidioides* in our gene family analysis (PAAG_02977/PABG_00438/PADG_02849); this gene is related to lovastatin nonaketide synthase genes present in all fungi. Compared to other filamentous Ascomycetes, *Paracoccidioides* has a small number of secondary metabolite genes, and lacks typical classes of enzymes including terpene synthases and dimethylallyl tryptophan synthases [70].

Genes that regulate SMGCs in related fungi are also conserved in *Paracoccidioides* (Table S15). In *Aspergillus* species the expression of several SMGCs are coordinately regulated by a single protein, LaeA [71], [72]. In *H. capsulatum*, two homologs of Velvet A, Ryp2 and Ryp3, and a transcription factor, Ryp1, were shown to be required for yeast phase growth [73]; orthologs found in *Paracoccidioides* may play similar roles (Table S16). Since *H. capsulatum* Ryp2 and Ryp3 are essential for viable spore production and regulation of sporulation at room temperature, the Velvet A complex in dimorphic fungi may connect temperature signaling to development.

### Identification of potential drug targets in *Paracoccidioides*

The targets of known antifungal drugs are conserved in *Paracoccidioides*, and comparative analysis identifies additional potential targets. The most common target of antifungal drugs, including azoles and allylamines, is the ergosterol biosynthesis pathway. Ergosterol is the major sterol in fungal cell membranes, and is important for fungal membrane fluidity and permeability. As it is not found in mammalian cells, drugs that target ergosterol biosynthesis specifically are selective. Genes involved in sterol biosynthesis are highly conserved in the three *Paracoccidioides* genomes (Text S1 part 5, Table S17). Among them, the gene encoding sterol 24-C-methyltransferase (*SMT*) has been investigated as a drug target. Studies identified the azasterol analogs AZA-1, AZA-2, AZA-3 [74] and 25-azalanosterol [75] as potent inhibitors of this enzyme in *P. brasiliensis*.

Cellular host immune responses and antifungal drugs also target fungal chitin and glucan components of the cell wall and associated proteins [76]. Another class of drugs, echinocandins, target β-1,3-D-glucan synthase which is essential for assembling a functional cell wall. *P. brasiliensis* is susceptible to the echinocandin caspofungin (up to 1 µg/ml, with sensitivity varying between isolates and morphological phase [77]; mycelia, which contain mostly β-1,3-glucan are more sensitive than yeast, which contain mostly α-1,3-glucan [78], [79]. Each of the *Paracoccidioides* genomes contains seven chitin synthases, one of each of the seven fungal chitin synthase classes (Table S18). This includes the first identification of a class III chitin synthase in a dimorphic fungus; this class was previously reported only in filamentous fungi [80], but is conserved in all species in our gene family analysis. To identify additional potential drug targets, we identified a total of 1,977 proteins that are highly conserved in *Paracoccidioides*, but not found in the human genome (Text S1 part 6, Table S19). In addition to proteins important for cell wall function, metabolic enzymes from pathways absent in humans can be useful targets for the development of new drugs.

## Discussion

In this study we sequenced the genomes of three strains of the pathogenic fungal genus *Paracoccidioides* and characterized the shared and unique gene content compared to related dimorphic and non-dimorphic fungi. This uncovered a reduced content for many genes, including those involved in carbohydrate metabolism, protein catabolism, and synthesis of secondary metabolites within the Onygenales, including *Paracoccidioides*. Given the added complexity of maintaining a dimorphic lifestyle, this reduction in gene content seems unexpected. The ability to produce a different morphological form which lives in a different environment and faces different environmental pressures suggests that novel genes would need to evolve in concert. The lack of gene family expansions suggests that genes already present in the ancestors of the dimorphic fungi gained additional functions, perhaps through more complicated regulation. This could explain the rapid evolution of transcription factors within *Paracoccidioides*, which could allow changes in gene regulation in these species.

Despite a reduced number of proteases in the Onygenales compared to their relatives, protein metabolism appears to be central to growth in the Onygenales. Metabolic assays of the non-pathogenic Onygenales *Uncinocarpus reesii* showed extensive growth on a wide range of proteinaceous substrates compared to growth on a very limited range of carbohydrates, namely cellulose and its component glucose. These results suggest that in the soil environment where they are commonly found, Onygenales are likely saprophytes that digest both cellulosic plant materials and a wide range of animal proteins. Furthermore, the superior growth of *U. reesii* on proteinaceous substrates relative to carbohydrates may predispose Onygenales to a lifestyle as an animal pathogen. For example, *Paracoccidioides* has been shown to secrete a serine protease capable of degrading the basement membrane, and potentially playing a role in host invasion [81]. These results also contrast with those found for *Trichoderma reesei*, a filamentous ascomycete used as an industrial degrader of plant biomass, which show substantially higher growth rates on carbohydrates than proteins.

Despite showing contractions of numerous gene families, *Paracoccidioides* contain expanded numbers of FunK1 fungal-specific kinases. Since this family is only present in multicellular fungi, it has been hypothesized to potentially play a role in fungal multicellularity [38]. We did not find FunK1 genes in several multicellular fungi in our comparative data set, notably *Aspergillus* and *Neurospora*. Therefore, while these genes may play a role in fungal multicellular processes, it is unlikely that they are required for multicellular growth. Regardless, given the distinctiveness of FunK1 genes from other protein kinases, further experiments will need to be conducted to elucidate the functions of FunK1 genes.

In total these data provide new insights into the gene repertoire and physiological potential of dimorphic fungi and *Paracoccidioides* in particular. Additionally, the genomic resources presented here, including the genome sequences and annotations, the optical map, and the large SNP dataset, will allow further investigation into the genetic basis of morphogenesis and pathogenicity in *Paracoccidioides* and the dimorphic fungi.

## Materials and Methods

### Selection of isolates for sequencing

Three isolates were chosen for whole genome sequencing. Pb01 is a *P. lutzii* clinical isolate from an acute form of paracoccidioidomycosis in an adult male. Pb03 is a *P. brasiliensis* isolate from chronic PCM and represents the PS2 phylogenetic group. Pb18 is a *P. brasiliensis* isolate from chronic PCM and represents the S1 phylogenetic group. Pb18 is virulent in mice when inoculated by the intraperitoneal, intratracheal and intravenous routes [82], while Pb03 (and other PS2 isolates) was comparatively less virulent in B10.A mice [20]. Some phenotypic differences of Pb03 with Pb18 were also suggested by differences in transcriptional regulation in heat shock genes in Pb03 when compared with Pb18 [22].

### Sequencing, assembly, and annotation

Three whole genome shotgun sequencing libraries were constructed from genomic DNA for each strain; two plasmid libraries (4 kb and 10 kb inserts) and a Fosmid library (40 kb inserts). Paired end-sequence was generated for each with Sanger technology (Table S20), and assembled using Arachne [30]. The assemblies range in size from 29 to 33 Mb, in agreement with previous genome size estimates of 30 Mb for each strain by pulsed-field gel electrophoresis (PFGE) [24], [83]. Between 95–97% of reads were assembled for each genome, resulting in 8.0X (*P. lutzii*), 8.9X (Pb03), and 9.8X (Pb18) assemblies. Mitochondrial scaffolds were identified based on comparison to the published sequence for Pb18 [34] and separated out. To identify telomeric sequence, we searched for arrays of the telomeric repeat (TTAGGG) in the assembly as well as the unassembled reads (Text S1 part 1). The assemblies and annotations for the three *Paracoccidioides* genomes were deposited in GenBank under the following accession identification numbers: *Paracoccidioides lutzii* Pb01 (ABKH00000000), *Paracoccidioides brasiliensis* Pb03 (ABHV00000000), and *Paracoccidioides brasiliensis* Pb18 (ABKI00000000).

Protein coding genes in the *P. brasiliensis* and *P. lutzii* genomes were identified by a combination of EST-based transcript identification, computational gene prediction programs, and manual revision of flagged calls (Text S1 part 2). A set of 41,463 ESTs were retrieved from GenBank at the start of the project; these include several experimental conditions including yeast phase [54], [56], mycelium and yeast cells [84], as well as additional unpublished sequences. Genes that did not fall into an orthologous group with any gene in the 15 taxon fungal data set (see evolutionary analysis section below), without a BLASTP hit of 1e-10 or lower against the GenBank non-redundant protein database, without a HMMER PFAM domain match of 1e-10 or lower, and without representative ESTs were flagged as likely false positive genes, categorized as dubious gene predictions. The initial gene sets were also filtered to remove transposable elements (Text S1 part 2). Every annotated gene was given a locus number of the form PAAG_##### (*P. lutzii*), PABG_##### (Pb03), PADG_##### (Pb18) which serves as a unique identifier within each genome and across assemblies. Secreted proteins were predicted using the hidden markov model (HMM) in Signalp [49] with a signal peptide probability cutoff of 0.9. GPI-anchored proteins were predicted using PredGPI [85] with a false positive rate <0.005. Mitochondrial genomes for each strain were separated from the nuclear assembly and annotated separately (Text S1 part 2).

### Chromosome anchoring of Pb18 assembly

To anchor the assembly for Pb18 onto chromosomes, we constructed a physical map for this strain. We used optical mapping, which is a single-molecule approach for the construction of ordered restriction maps [86]. An optical map for *P. brasiliensis* Pb18 was constructed by OpGen (www.opgen.com) using the restriction enzyme BsiWI (ĈGTACG). The map includes ∼100X physical coverage and consists of five linkage groups, suggesting five chromosomes. This is supported by a previous estimate by PFGE of four or five chromosomes [83], by flush ends to molecules found at the end of each map (data not shown), and by telomeric linkage for seven of the ten terminal scaffold ends (Text S1 part 1). To align the assembly to the map, *in silico* restriction maps of the genome assembly were generated. The correspondences of the restriction enzyme cutting sites and the predicted fragment lengths were used to order and orient the scaffolds on the optical map. A total of 19 scaffolds covering 28.14 Mb (94.0% of the assembly) were mapped to five linkage groups (Figure 1, Table S1). The remaining 38 scaffolds totaling 1.81 Mb in size did not anchor to the assembly. This sequence is highly repetitive; whereas the 9.2% of the Pb18 assembly consists of repeats, 54.5% of the unanchored scaffolds consist of repeats.

### Whole genome alignments

Genome assemblies were aligned using MUMmer [87]. Nucmer and promer were run at default settings, with the exception of using 1-to-1 alignment parameters allowing for rearrangements (Table S2, Figure S1). Mitochondrial genomes were aligned using nucmer, requiring unique matches (-mum option). DAGChainer [31] was used to identify syntenic regions, requiring blocks of at least 6 paired genes.

### Identification of genome-wide polymorphisms

SNPs were identified between the *Paracoccidioides* strains by comparing the sequencing reads from the *P. brasiliensis* Pb03 and *P. lutzii* genomes with the *P. brasiliensis* Pb18 assembly. Pb03 and *P. lutzii* sequencing reads were aligned with the Pb18 assembly using Blat, filtered for unique placement, and SNPs identified based on Neighborhood Quality Standard parameters (Text S1 part 3). SNPs were submitted to dbSNP at NCBI and released in build id 133, with rs# 154774690-155410572. This data can be accessed at http://www.ncbi.nlm.nih.gov/SNP/snp_viewTable.cgi?handle=BROAD-GENOMEBIO.

### Analysis of repetitive elements

*Paracoccidioides* genomes were scanned for transposable elements with four programs. Two tools, LTRharvest [88] and LTR_FINDER [89], are dedicated to identification of LTR retroelements; both programs search for LTR TE specific features such as long terminal repeats. TransposonPSI, which is an application of PSI-BLAST, was used with the distributed set of curated profiles for conserved ORFs present in different transposable elements (http://transposonpsi.sourceforge.net). RepeatModeler followed by RepeatMasker 3.2.7 was run with the RepBase library of manually curated mobile element and repetitive DNA sequences as templates [90], with original TE classification [91] applied.

To search for protein domains, all mobile elements were translated in all six frames using the transeq tool from EMBOSS suite [92] which were analyzed with HMMer3 [93], followed by protein domain searches against PFAM database [94] using the pfam_scan.pl script from the Pfam web page, to identify genes specific to different TE classes. DNA, LTR retrotransposons and LINE elements from each genome were clustered into families with CD-HIT [95], a fast clustering tool based on word counting algorithms, with the threshold of 80% identity (word length 5, identity 80%, shorter sequence coverage 90%).

Transposase_5 translated sequences from the three *Paracoccidioides* genomes were clustered with CLANS, a java based application based on PSI-BLAST, with a p-value of 0.01 [96]. The protein sequences detected with HMMer3 correspond to the central region of the Transposase_5 domain, but they are not full length. The CLANS clustering of all *Paracoccidioides* Transposase_5 sequences (Figure S4) identifies one group specific to*P. lutzii*, one conserved between *P. lutzii* and Pb18, and one common to all *Paracoccidioides*. A Pb03 element is also found in a unique class, but this sequence is highly degenerate and interrupted by stop codons. A cluster analysis with all reference Transposase_5 sequences from the Pfam 24 database (Figure S5) shows that the *P. lutzii* element is more closely related to Tc1 elements than Mariners.

Simple and low complexity repeats were identified using RepeatMasker (version open-3.2.5).

### Functional classification and analysis

Genes were functionally annotated using BLAST2GO [39], with a minimum e-value of 1×10^−10^. Fisher's exact test was used to detect enrichment of GO terms between groups of interest. We further annotated carbohydrate active enzymes and peptidases using the CAZY [40] and MEROPS [46] databases, respectively. Proteins were searched against their corresponding databases using BLAST [97], with minimum e-values of 1×10^−80^ for CAZY and 1×10^−20^ for MEROPS. Statistical comparisons of dimorphic versus non-dimorphic carbohydrate active enzymes and peptidases were made using Mann-Whitney U tests.

Metabolic pathways were characterized using Pathway Tools [44]. Metabolic reconstruction was performed using EFICAz2 [98] to assign Enzyme Commission (EC) (http://www.chem.qmul.ac.uk/iubmb/enzyme/) numbers for each enzyme. EC numbers and gene names were used as input to the Pathologic software with transport-identification-parser and pathway-hole-filler options set to assign biocyc pathways for each organism [99]. The full set of metabolic pathways for each genome, along with detailed reports of pathway evidence, pathway holes filled, transporters predicted, and EFICAz2 evidence and probabilities, are available at FungiCyc (http://fungicyc.broadinstitute.org).

### Evolutionary analysis

Genomes of Onygenales and other Ascomycetes were compared, including the three *Paracoccidioides* species as well as the following: *Histoplasma capsulatum* NAm1 (AAJI00000000), *Coccidioides immitis* RS (AAEC00000000), *Coccidioides posadasii* C735 delta SOWgp (ACFW00000000), *Microsporum gypseum* CBS 118893 (ABQE00000000), *Uncinocarpus reesii* 1704 (AAIW00000000), *Aspergillus nidulans* FGSC A4 (AACD00000000), *Aspergillus flavus* NRRL3357 (AAIH00000000), *Aspergillus fumigatus* Af293 (AAHF00000000), *Magnaporthe grisea* 70-15 (AACU00000000), *Neurospora crassa* OR74A (AABX00000000), *Fusarium oxysporum* f. sp. lycopersici 4287 (AAXH00000000), and *Penicillium chrysogenum* Wisconsin 54-1255 (NS_000201). Genes were clustered using OrthoMCL with a Markov inflation index of 1.5 and a maximum e-value of 1×10^−5^ [100]. We constructed two sets of gene clusters: one from the full set of 15 taxa and one from a reduced set including the three *Paracoccidioides* species and *H. capsulatum*. A table of the genes in each cluster for the full set of 15 taxa is available on the Broad *Paracoccidioides* website (http://www.broadinstitute.org/annotation/genome/paracoccidioides_brasiliensis/MultiDownloads.html).

To estimate a phylogeny we used 309 clusters that were present as single copies in all genomes. These clusters were then aligned using MUSCLE [101] and poorly aligned regions were trimmed using GBLOCKS under default settings [102]. We then estimated a phylogeny using the PROTGAMMABLOSUM62 model in RAxML [103] with 1,000 bootstrap replicates.

To study evolutionary rates within the *Paracoccidioides* and *Histoplasma* lineage, we identified all single copy orthologs present in all 4 genomes in the reduced ortholog set. Clusters were aligned using MUSCLE [101]. We then calculated dN/dS for all clusters which shared >80% length using the CODEML program of the PAML package (version 3.15) [104] using the relevant topology from the phylogenetic analysis. To identify GO terms representing categories of rapidly evolving genes, we resampled dN/dS without replacement onto genes to generate 1000 pseudoreplicates. For each GO term, we then compared the observed distribution of dN/dS to the resampled distribution to calculate a p-value. P-values were then corrected for multiple comparisons using the false discovery rate of Storey [105]. Ancestral character state reconstruction was performed using GeneTRACE [41].

### Catabolic phenotype screen

Metabolic analysis was conducted on *Uncinocarpus reesii*, a non-pathogenic soil dwelling fungus in the Onygenales. *U. reesii* is the closest non-pathogenic relative of *Paracoccidioides* for which a complete genome sequence is available. *U. reesii* strain UAMH 3881 was purified to single colonies on agar plates containing ½ Yedex at room temperature. To confirm the species of the culture, genomic DNA was extracted by bead beating in phenol and the ITS region was amplified using ITS1F [106] and ITS4 [107] primers and sequenced by Sanger technology (GenBank accession JF451137). By comparing to other sequences in the GenBank nt nucleotide database using MEGABLAST, the closest matches were identified as other *Uncinocarpus reesii* ITS sequences from other strains, at 97–99% identity and 99–100% coverage. After growth for 1 week, spores were gathered using sterile cotton swabs by scraping from the surface of growth on the plate. Cells were innoculated into 12 ml FF innoculating fluid (Biolog, Hayward CA). The resuspension of cells was gently vortexed to disaggregate the cell mass, then spun at 5000 x G for four minutes to separate clumps from well-dispersed innoculum. Supernatants were diluted with another 12 ml and mixed by inversion. 100 µl of resuspension was innoculated into PM1 and PM2a plates in triplicate (Biolog, Hayward CA). Growth was monitored at days 1, 3, 5, and 7 by measuring optical density at 590 nm of *U. reesii* growing on a compound by subtracting average OD590 values of the values for compounds without cells, as well as subtracting the average absorbance values for negative controls that contain cells without carbon sources. The Biolog system measures absorbance at 590 nm, and does not measure colors typical of secondary metabolites, such as red, orange, or brown. Furthermore, in the fungal-specific system, the experiment only measures turbidity rather than in bacterial experiments where 590 nm is measured in the presence of growth sensing purple dyes. Values above 0.15 after background subtraction at day 7 were taken as evidence of growth that was further supported by kinetic increases in the light scattering measurements during the time course. The growth substrates that were identified from the 190 compounds screened were separated into categories containing nitrogenous compounds, carbohydrates and oligosaccharides.

### FunK1 analysis

Orthologous groups of protein kinases identified as unique to the dimorphic fungi were identified as FunK1 genes and then aligned using MUSCLE [101]. This alignment was subsequently used with Hmmer [108] to identify additional FunK1 genes. The final set of FunK1 genes was realigned with MUSCLE and visualized with WebLogo [109].

### Secondary metabolism genes

We predicted secondary metabolite gene clusters (SMGC) using the Secondary Metabolite Unique Regions Finder (SMURF; http://www.jcvi.org/smurf/index.php). Ten backbone enzymes were found in each of the *Paracoccidioides* genomes, and all are conserved as single copy orthologs, found in syntenic regions between the genomes.

Within each genome, two classes of enzymes were found; a total of six nonribosomal peptide synthases (NRPSs) and four polyketide synthases (PKSs) (Table S15). No dimethylallyl tryptophan synthases (DMATSs) or and terpene cyclases (TCs) were identified. Two clusters are closely linked in the *P. brasiliensis* genomes; one is a cluster around a NRPS (PABG_00426/PADG_02836) and the other around a PKS (PABG_00438/PADG_02849).

### Drug target search

To filter genes conserved in humans, *Paracoccidioides* protein sequences from *P. lutzii*, Pb03 and Pb18 were queried using BLAST against a human genome protein sequence database (HG17, Build 35) (Filter query sequence =  False, expect  = 0.001). *P. brasiliensis* sequences that did not hit a human sequence were mapped to conserved orthoMCL orthologs in *Paracoccidioides*. This initial analysis identified 1,977 genes, of which 1,628 (82.4%) genes were named as conserved hypothetical protein, hypothetical protein or predicted proteins indicating a high fraction of poorly conserved or dubious genes. From those genes, 38 genes were selected (Table S18), which were present in the three *Paracoccidioides* isolates, based on their potential as drug targets based on localization or conservation in other pathogens. These function of these genes and support for their potential as targets is described in the supplement (Text S1 part 6).

## Supporting Information

Figure S1 Dotplot views of mummer genome alignments. A. Nucmer alignments of *Paracoccidioides* genomes. B. Promer alignments of *Paracoccidioides* genomes to *H. capsulatum* and *C. immitis*.
(EPS)

Figure S2 Distribution of SNPs for *P. lutzii* (red) and *P. brasiliensis* (Pb03) (blue) across *P. brasiliensis* (Pb18) chromosomes. Pb18 supercontigs are shown as grey shaded rectangles at the top of each chromosome plot.
(EPS)

Figure S3 Transposable element composition of the *Paracoccidioides* genomes.
(EPS)

Figure S4 CLANS clustering of *Paracoccidioides* Transposase_5 domain Tc1 elements.
(EPS)

Figure S5 CLANS clustering of Transposase_5 domain Tc1 elements.
(EPS)

Table S1 Pb18 optical map and assembly alignment statistics.
(DOC)

Table S2 Nucmer genome alignment statistics.
(DOC)

Table S3 Gene Ontology (GO) and PFAM terms enriched in unique regions of the *Paracoccidioides* genomes.
(DOC)

Table S4 Simple and low complexity repeats content of genomes.
(DOC)

Table S5 Mitochondrial genome statistics.
(DOC)

Table S6 Gene Ontology (GO) terms significantly over- or under-represented.
(XLS)

Table S7 CAZY carbohydrate active enzyme classification counts for 15 fungal genomes.
(XLS)

Table S8 MEROPS peptidase classification counts for 15 fungal genomes.
(XLS)

Table S9 Degradation pathways for amino acids and carbohydrates in *Paracoccidioides* and *Uncinocarpus*.
(DOC)

Table S10 Carbon source utilization by *Uncinocarpus reesii*.
(XLS)

Table S11 Predicted Histidine kinases.
(DOC)

Table S12 Rapidly evolving Gene Ontology (GO) terms in selected groups.
(DOC)

Table S13 Conservation of mating genes.
(XLS)

Table S14 Conservation of meiosis genes.
(XLS)

Table S15 Predicted secondary metabolite backbone enzymes.
(DOC)

Table S16 Homologs of VelvetA complex, LaeA, and Ryp1.
(DOC)

Table S17 Conservation of genes involved in sterol biosynthesis.
(DOC)

Table S18 Chitin synthases.
(DOC)

Table S19 Potential drug targets conserved in *Paracoccidioides*.
(DOC)

Table S20 Sequence statistics for trimmed reads.
(DOC)

Text S1 Supplementary methods and text.
(DOC)
