# Supplementary material for: Comparative Genomic Analysis of Human Fungal Pathogens Causing Paracoccidioidomycosis
Source: PLoS Genet. 2011 Oct 27;7(10):e1002345. doi: 10.1371/journal.pgen.1002345 (PMC3203195; doi:10.1371/journal.pgen.1002345)
Supplement: Table S1 — Pb18 optical map and assembly alignment statistics. (DOC) [file pgen.1002345.s006.doc]

**Table S1. Pb18 optical m**ap and assembly alignment statistics.

| Optical linkage group | Linkage group length (Mb) | Mapped scaffolds | Total scaffold size (Mb) |
| --- | --- | --- | --- |
| 1 | 10.50 | 1,11,7,3 | 10.18 |
| 2 | 5.89 | 9,6,17,15,5 | 6.07 |
| 3 | 5.40 | 26,2,13 | 4.60 |
| 4 | 4.47 | 12,4,18 | 4.26 |
| 5 | 3.20 | 10,14,8,16 | 3.03 |
| Total | 29.47 |  | 28.14 |
| Unmapped scaffolds | - | 19-25,27-57 | 1.81 |
