# Supplementary material for: Comparative Genomic Analysis of Human Fungal Pathogens Causing Paracoccidioidomycosis
Source: PLoS Genet. 2011 Oct 27;7(10):e1002345. doi: 10.1371/journal.pgen.1002345 (PMC3203195; doi:10.1371/journal.pgen.1002345)
Supplement: Table S2 — Nucmer genome alignment statistics. (DOC) [file pgen.1002345.s007.doc]

**Table S2. Mum**mer genome alignment statistics.

| Reference genome | Query genome | Alignment method | Number of alignment blocks | Total alignment length(kb) | Average percent identity |
| --- | --- | --- | --- | --- | --- |
| *P. lutzii* | *P. brasiliensis Pb18* | Nucmer | 5,426 | 24,404 | 90.2 |
| *P. lutzii* | *P. brasiliensis Pb03* | Nucmer | 5,283 | 24,317 | 90.2 |
| *P. lutzii* | *H. capsulatum* Nam1 | Nucmer | 1,062 | 836 | 86.8 |
| *P. lutzii* | *C. immitis* RS | Nucmer | 127 | 91 | 88.0 |
| *P. brasiliensis Pb03* | *P. brasiliensis Pb18* | Nucmer | 2,165 | 27 | 96.4 |
| *P. brasiliensis Pb18* | *H. capsulatum* Nam1 | Nucmer | 959 | 729 | 86.7 |
| *P. brasiliensis Pb18* | *C. immitis* RS | Nucmer | 122 | 72 | 88.3 |
| *P. brasiliensis Pb18* | *H. capsulatum* Nam1 | Promer | 1,240 | 2,366 | 81.6 |
| *P. brasiliensis Pb18* | *C. immitis* RS | Promer | 504 | 1,049 | 79.8 |
