# Supplementary material for: Comparative Genomic Analysis of Human Fungal Pathogens Causing Paracoccidioidomycosis
Source: PLoS Genet. 2011 Oct 27;7(10):e1002345. doi: 10.1371/journal.pgen.1002345 (PMC3203195; doi:10.1371/journal.pgen.1002345)
Supplement: Table S3 — Gene Ontology (GO) and PFAM terms enriched in unique regions of the Paracoccidioides genomes. (DOC) [file pgen.1002345.s008.doc]

**Table S3. Gene Ontology (GO) and PFAM terms significantly enriched in unique (non-syntenic) regions of the 3 *Paracoccidiodes* genomes. P-values were calculated using Fisher’s exact test and Bonferroni corrected.**

| Term ID | # in unique regions | # in syntenic regions | P-value | Term description |
| --- | --- | --- | --- | --- |
| GO:0000785 | 23 | 45 | 2.45E-08 | chromatin |
| GO:0000786 | 15 | 24 | 1.37E-05 | nucleosome |
| GO:0003676 | 312 | 2169 | 2.13E-36 | nucleic acid binding |
| GO:0003964 | 12 | 3 | 3.67E-09 | RNA-directed DNA polymerase activity |
| GO:0004310 | 4 | 0 | 0.0428 | farnesyl-diphosphate farnesyltransferase activity |
| GO:0004523 | 17 | 5 | 1.69E-13 | ribonuclease H activity |
| GO:0004713 | 17 | 22 | 1.18E-07 | protein tyrosine kinase activity |
| GO:0006278 | 11 | 6 | 1.35E-06 | RNA-dependent DNA replication |
| GO:0006323 | 15 | 38 | 0.00148 | DNA packaging |
| GO:0006334 | 15 | 32 | 0.000255 | nucleosome assembly |
| GO:0006468 | 76 | 395 | 3.85E-11 | protein amino acid phosphorylation |
| GO:0008270 | 163 | 1228 | 1.83E-12 | zinc ion binding |
| GO:0015074 | 26 | 3 | 3.19E-25 | DNA integration |
| GO:0031497 | 15 | 32 | 0.000255 | chromatin assembly |
| PF00069 | 52 | 362 | 1.03E-11 | Pkinase |
| PF00075 | 7 | 2 | 7.45E-06 | RnaseH |
| PF00098 | 35 | 46 | 7.64E-26 | zf-CCHC |
| PF00125 | 15 | 34 | 2.71E-07 | Histone |
| PF00432 | 14 | 23 | 4.33E-08 | Prenyltrans |
| PF00665 | 17 | 0 | 6.63E-22 | rve |
| PF00786 | 4 | 0 | 0.00489 | PBD |
| PF01036 | 4 | 0 | 0.00489 | Bac_rhodopsin |
| PF01184 | 6 | 0 | 6.20E-06 | Grp1_Fun34_YaaH |
| PF02755 | 9 | 0 | 2.77E-10 | RPEL |
| PF03184 | 37 | 3 | 3.76E-47 | DDE |
| PF04095 | 6 | 0 | 6.20E-06 | NAPRTase |
| PF07727 | 5 | 0 | 0.000174 | RVT_2 |
| PF10236 | 4 | 0 | 0.00489 | DAP3 |
| PF11917 | 25 | 30 | 1.64E-18 | DUF3435 |
| PF00069 | 52 | 362 | 1.03E-11 | Pkinase |
