# Supplementary material for: Comparative Genomic Analysis of Human Fungal Pathogens Causing Paracoccidioidomycosis
Source: PLoS Genet. 2011 Oct 27;7(10):e1002345. doi: 10.1371/journal.pgen.1002345 (PMC3203195; doi:10.1371/journal.pgen.1002345)
Supplement: Table S4 — Simple and low complexity repeats content of genomes. (DOC) [file pgen.1002345.s009.doc]

**Table S4**. Simple and low complexity repeats content of genomes.

|  | Total bases | | | Percent of genome | | |
| --- | --- | --- | --- | --- | --- | --- |
|  | *P. lutzii* | Pb03 | Pb18 | *P. lutzii* | Pb03 | Pb18 |
| Simple repeats | 417,430 | 342,908 | 367,421 | 1.27% | 1.18% | 1.23% |
| Low complexity | 842,793 | 576,231 | 589,503 | 2.56% | 1.98% | 1.97% |
| AT-rich low complexity | 678,392 | 425,712 | 441,474 | 2.08% | 1.48% | 1.50% |
