# Supplementary material for: Comparative Genomic Analysis of Human Fungal Pathogens Causing Paracoccidioidomycosis
Source: PLoS Genet. 2011 Oct 27;7(10):e1002345. doi: 10.1371/journal.pgen.1002345 (PMC3203195; doi:10.1371/journal.pgen.1002345)
Supplement: Table S5 — Mitochondrial genome statistics. (DOC) [file pgen.1002345.s010.doc]

**Table S5**. Mitochondrial genome statistics.

|  | *P. lutzii* | Pb03 | Pb18* |
| --- | --- | --- | --- |
| Total scaffold size (Kb) | 40.7 | 75.2 | 71.3 |
| Total contig Length (Kb) | 30.7 | 73.9 | 71.1 |
| Scaffolds | 1 | 1 | 1 |
| Contigs | 2 | 7 | 3 |
| GC content (%) | 23.1 | 21.0 | 21.1 |
|  |  |  |  |
| Protein-coding genes | 12 | 16 | 18 |
| tRNAs | 26 | 32 | 25 |
|  |  |  |  |
| *Statistics from previous work [9] |  |  |  |
