# Supplementary material for: Comparative Genomic Analysis of Human Fungal Pathogens Causing Paracoccidioidomycosis
Source: PLoS Genet. 2011 Oct 27;7(10):e1002345. doi: 10.1371/journal.pgen.1002345 (PMC3203195; doi:10.1371/journal.pgen.1002345)
Supplement: Table S9 — Degradation pathways for amino acids and carbohydrates in Paracoccidioides and Uncinocarpus. (DOC) [file pgen.1002345.s014.doc]

**Table S9. Degradation pathways for amino acids and carbohydrates in *Paracoccidioides* and *Uncinocarpus***.

| Degradation Pathway | *Uncinocarpus* | *Paracoccidioides* | *Coccidioides* | *Histoplasma* |
| --- | --- | --- | --- | --- |
| Amino Acids |  |  |  |  |
| Alanine | P | P | P | P |
| Arginine | P | P | P | P |
| Asparagine | A | P | P | P |
| Aspartate | P | P | P | P |
| Cysteine | P | P | P | P |
| Glutamate | P | P | P | P |
| Glutamine | P | P | P | P |
| Glycine | P | P | P | P |
| Histidine | A | A | A | A |
| Homocysteine | A | A | A | A |
| Homoserine | A | A | A | A |
| Isoleucine | P | P | P | P |
| Leucine | P | P | P | P |
| Lysine | P | P | P | P |
| Methionine | P | P | P | P |
| Ornithine | P | A | P | A |
| Phenylalanine | P | P | P | P |
| Proline | P | P | P | P |
| Serine | P | P | P | P |
| Threonine | A | P | A | A |
| Tryptophan | P | P | P | P |
| Tyrosine | P | P | P | P |
| Valine | P | P | P | P |
| Carbohydrates |  |  |  |  |
| Arabinose | A | A | A | A |
| Galactose | A | P | A | P |
| Lactose | A | A | A | P |
| Sucrose | A | A | A | A |
| Trehalose | P | P | P | P |
| Xylose | P | P | P | P |
| Glucose | P | P | P | P |
| Fructose | P | P | P | P |
| Mannose | P | P | P | P |
| Meliobiose | A | P | A | P |
