# Supplementary material for: Comparative Genomic Analysis of Human Fungal Pathogens Causing Paracoccidioidomycosis
Source: PLoS Genet. 2011 Oct 27;7(10):e1002345. doi: 10.1371/journal.pgen.1002345 (PMC3203195; doi:10.1371/journal.pgen.1002345)
Supplement: Table S11 — Predicted Histidine kinases. (DOC) [file pgen.1002345.s016.doc]

**Table S11. Predicted Histidine kinases in *Paracoccidioides*** genomes.

| Type | *S. cerevisiae* | *S. pombe* | *C. albicans* | *A. nidulans* | *H. capsulatum* | *P. brasiliensis* Pb03 | *P. brasiliensis* Pb18 | *P. lutzii* |
| --- | --- | --- | --- | --- | --- | --- | --- | --- |
| Sensor Kinase | Sln1p | Mak1 | CaSln1 | AN3101.3 | HCAG_06665 | PABG_02482 | PADG_00903 | PAAG_07139^ |
|  |  |  |  | AN4447 | HCAG_04184 | PABG_01462 | PADG_04063 | PAAG_00767 |
|  |  | Mak2 | CaHk1 | AN3102.3 | HCAG_06649 | PABG_02483 | PADG_00905 | PAAG_07142^ |
|  |  |  |  | AN5296.1 (TcsA) | none | PABG_05147 | PADG_07139 | PAAG_00121 |
|  |  | Mak3 | CaNik1 | AN4479.3 | HCAG_04502 | PABG_06372 | PADG_07579 | PAAG_05810# |
| Histidine-containing phosphotransfer intermediate (HPt) | Ypd1 | Ydp1 | CaYpd1 | AN2005.3 (YpdA) | HCAG_06099 | PABG_03087 | PADG_01617 | PAAG_02599^ |
| Response regulator | Skn7 | Prr1 | CaSkn7 | AN3688.3 (SrrA) | HCAG_05282 | PABG_04520 | PADG_04915 | PAAG_05064^ |
|  | Ssk1p | Mcs4 | CaSsk1 | AN7697.3 (SskA) | HCAG_00732 | PABG_03853 | PADG_07294 | PAAG_04020,PAAG_04021*^ |

**^single copy orthologs in the 15 genomes analyzed for gene families. #orthologs and paralogs in the 15 genomes analyzed *gene split between these loci.**
