# Supplementary material for: Comparative Genomic Analysis of Human Fungal Pathogens Causing Paracoccidioidomycosis
Source: PLoS Genet. 2011 Oct 27;7(10):e1002345. doi: 10.1371/journal.pgen.1002345 (PMC3203195; doi:10.1371/journal.pgen.1002345)
Supplement: Table S12 — Rapidly evolving Gene Ontology (GO) terms in selected groups. (DOC) [file pgen.1002345.s017.doc]

**Table S12.** **Rapidly evolving GO terms in the *Paracoccidioides* and *Histoplasma* lineage.**

| GO ID | GO Term | Q-value* |
| --- | --- | --- |
| GO:0008270 | Zinc ion binding | 0 |
| GO:0003700 | Transcription factor activity | 0 |
| GO:0006355 | Regulation of transcription, DNA-dependent | 0 |
| GO:0043565 | Sequence-specific DNA binding | 0 |
| GO:0030001 | Metal ion transport | 0.041 |

* GO terms with significant q-values, p-values corrected for multiple comparisons using the false discovery rate [52], are shown.
