# Supplementary material for: Comparative Genomic Analysis of Human Fungal Pathogens Causing Paracoccidioidomycosis
Source: PLoS Genet. 2011 Oct 27;7(10):e1002345. doi: 10.1371/journal.pgen.1002345 (PMC3203195; doi:10.1371/journal.pgen.1002345)
Supplement: Table S15 — Predicted secondary metabolite backbone enzymes. (DOC) [file pgen.1002345.s020.doc]

**Table S15. Predicted secondary metabolite backbone enzymes**.

| *P. lutzii* | Pb03 | Pb18 | SMURF prediction | cluster size |
| --- | --- | --- | --- | --- |
| PAAG_01883 | PABG_02158 | PADG_00557* | PKS | 24 |
| PAAG_01679 | PABG_03726 | PADG_00102 | NRPS | 8 |
| PAAG_01545 | PABG_03602 | PADG_00233 | NRPS-Like | 19 |
| PAAG_04835 | PABG_00176 | PADG_02575 | NRPS-Like | 15 |
| PAAG_07698 | PABG_00426 | PADG_02836 | NRPS | 13 |
| PAAG_02977 | PABG_00438 | PADG_02849 | PKS | 11 |
| PAAG_08527 | PABG_04670 | PADG_05295 | NRPS | 1 |
| PAAG_02228 | PABG_04742 | PADG_05367 | PKS-Like | 13 |
| PAAG_03500^ | PABG_06839 | PADG_06324 | PKS | 1 |
| PAAG_05434 | PABG_05947 | PADG_06784 | NRPS-Like | 11 |
| *gene model fused with adjacent gene | | |  |  |
| ^not identified by SMURF | | |  |  |
