# Supplementary material for: Comparative Genomic Analysis of Human Fungal Pathogens Causing Paracoccidioidomycosis
Source: PLoS Genet. 2011 Oct 27;7(10):e1002345. doi: 10.1371/journal.pgen.1002345 (PMC3203195; doi:10.1371/journal.pgen.1002345)
Supplement: Table S16 — Homologs of VelvetA complex, LaeA, and Ryp1. (DOC) [file pgen.1002345.s021.doc]

**Table S16**. Homologs of VelvetA complex, LaeA, and Ryp1.

| *A. nidulans* | *H. capsulatum* | *P. lutzii* | *P. brasiliensis* (Pb03) | *P. brasiliensis* (Pb18) |
| --- | --- | --- | --- | --- |
| VeA | *RYP2/RYP3* | PAAG_03070 | PABG_00531 | PADG_02949 |
| VosA | ------ | PAAG_02671 | PABG_03151 | PADG_01695 |
| VelB | ------ | PAAG_06081 | PABG_06575 | PADG_08037 |
| LaeA | ------ | PAAG_05395 | PABG_05982 | PADG_06744 |
|  | *RYP1* | PAAG_03579 | PABG_06919 | PADG_06243 |
