# Supplementary material for: Comparative Genomic Analysis of Human Fungal Pathogens Causing Paracoccidioidomycosis
Source: PLoS Genet. 2011 Oct 27;7(10):e1002345. doi: 10.1371/journal.pgen.1002345 (PMC3203195; doi:10.1371/journal.pgen.1002345)
Supplement: Table S17 — Conservation of genes involved in sterol biosynthesis. (DOC) [file pgen.1002345.s022.doc]

**Table S17. Conservation of genes involved in sterol biosynthesis.**

| Enzyme | Isolate | Transcript | Protein length (aa) |
| --- | --- | --- | --- |
| 3-[Hydroxy 3-methylglutaryl-CoA reductase](http://www.yeastgenome.org/cgi-bin/GO/goTerm.pl?goid=4420) (*HMG1*) | *P. lutzii* | PAAG_05791.1 | 1158 |
|  | Pb03 | PABG_06358.1 | 1144 |
|  | Pb18 | PADG_07596.1 | 1143 |
| Squalene epoxidase (monooxygenase) (*ERG1*) | *P. lutzii* | PAAG_08773.1 | 1434 |
|  | Pb03 | PABG_00295.1 | 1596 |
|  | Pb18 | PADG_02704.1 | 1566 |
| Cytochrome P450-dependent C14 demethylase (*ERG11*) | *P. lutzii* | PAAG_00827.1 | 603 |
|  | Pb03 | PABG_01406.1 | 526 |
|  | Pb18 | PADG_04003.1 | 524 |
| Sterol 24-C-  methyltransferase (*SMT*) | *P. lutzii* | PAAG_01575.1 | 378 |
|  | Pb03 | PABG_03630.1 | 378 |
|  | Pb18 | PADG_00204.1 | 378 |
| C-5 sterol desaturase (*ERG3*) | *P. lutzii* | PAAG_03651.1 | 360 |
|  | Pb03 | PABG_06972.1 | 360 |
|  | Pb18 | PADG_06181.1 | 360 |
| Delta 24,24,1 sterol methyl reductase (*SMR*) | *P. lutzii* | PAAG_07980.1 | 542 |
|  | Pb03 | PABG_03513.1 | 542 |
|  | Pb18 | PADG_02082.1 | 543 |
| 7-Dehydrocholesterol reductase / Delta 7-reductase (*DHCR7*) | *P. lutzii* | PAAG_04766.1 | 427 |
|  | Pb03 | PABG_00118.1 | 517 |
|  | Pb18 | PADG_02509.1 | 478 |
