# Supplementary material for: Comparative Genomic Analysis of Human Fungal Pathogens Causing Paracoccidioidomycosis
Source: PLoS Genet. 2011 Oct 27;7(10):e1002345. doi: 10.1371/journal.pgen.1002345 (PMC3203195; doi:10.1371/journal.pgen.1002345)
Supplement: Table S18 — Chitin synthases. (DOC) [file pgen.1002345.s023.doc]

**Table S18.** **Chitin synthases identified in the *Paracoccidioides* genome databases.** *P. brasiliensis* Chs2 (GenBank Y09231) and Chs4 (GenBank EF654132) [53,54]were used to search the *P. brasiliensis* protein database using BLASTP and the genomic sequence database using TBLASTN.

| *Paracoccidioides* genome | Transcript or supercontig | Chitin synthase Class | Observations |
| --- | --- | --- | --- |
| Pb18 | PADG_06479 | Class I | missing 3’ end |
|  | PADG_08636 | Class II |  |
|  | PADG_06438 | Class III |  |
|  | PADG_02603 | Class IV |  |
|  | PADG_07913 | Class V |  |
|  | PADG_02784 | Class VI |  |
|  | PADG_07911 | Class VII |  |
| Pb03 | PABG_07036 | Class I |  |
|  | PABG_07704 | Class II |  |
|  | PABG_07074 | Class III |  |
|  | PABG_00203 | Class IV |  |
|  | PABG_06458 | Class V |  |
|  | PABG_00373 | Class VI |  |
|  | PABG_06456 | Class VII |  |
| *P. lutzii* | PAAG_03342 | Class I |  |
|  | PAAG_08385 | Class II |  |
|  | PAAG_03391 | Class III | missing 3’ end |
|  | PAAG_04862 | Class IV |  |
|  | PAAG_00215 | Class V |  |
|  | PAAG_07751 | Class VI |  |
|  | Supercontig 1: 808183-812238 + | Class VII |  |
