# Supplementary material for: Comparative Genomic Analysis of Human Fungal Pathogens Causing Paracoccidioidomycosis
Source: PLoS Genet. 2011 Oct 27;7(10):e1002345. doi: 10.1371/journal.pgen.1002345 (PMC3203195; doi:10.1371/journal.pgen.1002345)
Supplement: Table S19 — Potential drug targets conserved in Paracoccidioides. (DOC) [file pgen.1002345.s024.doc]

**Table S19. Potential Drug Targets conserved in *Paracoccidioides*.**

| Gene Product | Category | *P. lutzii* | Pb03 | Pb18 |
| --- | --- | --- | --- | --- |
| alpha-1,3-glucan synthase mok11 | cell wall | PAAG_03297 | PABG_00726 | PADG_03169 |
| exo-beta-1,3-glucanase | cell wall | PAAG_03359 | PABG_07048 | PADG_06465 |
| endo-1,3(4)-beta-glucanase | cell wall | PAAG_03880 | PABG_00905 | PADG_03375 |
| glucanase | cell wall | PAAG_02542 | PABG_05882 | PADG_06864 |
| alpha 1,2-mannosyltransferase | cell wall | PAAG_05421 | PABG_05957 | PADG_06770 |
| alpha-1,6-mannosyltransferase Och1 | cell wall | PAAG_07836 | PABG_07755 | PADG_08248 |
| chitinase | cell wall | PAAG_03849 | PABG_00883 | PADG_03347 |
| dolichyl phosphate-D-mannose:protein O-D-mannosyltransferase | cell wall | PAAG_07032 | PABG_03036 | PADG_01561 |
| Erg25 ergosterol biosynthesis | cell wall | PABG_00691 | PADG_03128 | PAAG_03253 |
| Erg28 ergosterol biosynthesis | cell wall | PAAG_03696 | PABG_00752 | PADG_03199 |
| aspartate-semialdehyde dehydrogenase (ASADH) | metabolic pathway | PAAG_08065 | PABG_07306 | PADG_08376 |
| beta-N-acetylhexosaminidase | cell wall | PADG_06728 | PABG_05994 | PAAG_05380 |
| prephenate dehydratase | metabolic pathway | PAAG_07380 | PABG_05562 | PADG_05878 |
| 6,7-dimethyl-8-ribityllumazine synthase | metabolic pathway | PAAG_00851 | PABG_01387 | PADG_03983 |
| thioesterase superfamily | metabolic pathway | PAAG_06629 | PABG_02647 | PADG_01085 |
| adenosine deaminase | metabolic pathway | PAAG_00523 | PABG_06782 | PADG_02162 |
| GMP synthase | metabolic pathway | PAAG_02333 | PABG_04834 | PADG_05472 |
| dihydrofolate reductase | metabolic pathway | PADG_05351 | PAAG_08913 | PABG_07774 |
| alcohol dehydrogenase | metabolic pathway | PAAG_08248 | PABG_03450 | PADG_02018 |
| mitochondrial F1F0 ATP synthase subunit | metabolic pathway | PAAG_02265 | PABG_04772 | PADG_05402 |
| metallopeptidase | metabolic pathway | PAAG_07202 | PABG_02532 | PADG_00962 |
| ubiquitin-specific protease | metabolic pathway | PADG_04938 | PAAG_05092 | PABG_04542 |
| ubiquitin family protein | metabolic pathway | PAAG_05332 | PABG_06039 | PADG_06675 |
| cytochrome P450 | other | PAAG_01848 | PABG_02127 | PADG_00528 |
| carbonic anhydrase | other | PAAG_05716 | PABG_06289 | PADG_07674 |
| tyrosine phosphatase | other | PAAG_04037 | PABG_03867 | PADG_07313 |
| macrophage migration inhibitory factor | other | PAAG_01158 | PABG_01732 | PADG_03671 |
| phospholipase D | other | PAAG_02042 | PABG_02293 | PADG_00705 |
| ribonuclease P subunit | other | PAAG_04510 | PABG_04292 | PADG_04671 |
| PDZ domain (also known as DHR or GLGF domains) | other | PAAG_04650 | PABG_04416 | PADG_04809 |
| tyrosinase | other | PAAG_01024 | PABG_01611 | PADG_03805 |
| DNA-directed RNA polymerase | other | PAAG_00396 | PABG_06677 | PADG_02279 |
| RNA-directed RNA polymerase | other | PABG_03975 | PADG_07439 | PAAG_04185 |
| Acetyltransferase (GNAT) family | other | PADG_05962 | PAAG_06044 | PABG_05634 |
| catechol O-methyltransferase | other | PAAG_04909 | PABG_00243 | PADG_02644 |
| uricase | other | PAAG_01437 | PABG_01949 | PADG_00331 |
| lipase (class 3) | other | PAAG_00013 | PABG_05054 | PADG_07242 |
