# Supplementary material for: Comparative Genomic Analysis of Human Fungal Pathogens Causing Paracoccidioidomycosis
Source: PLoS Genet. 2011 Oct 27;7(10):e1002345. doi: 10.1371/journal.pgen.1002345 (PMC3203195; doi:10.1371/journal.pgen.1002345)
Supplement: Table S20 — Sequence statistics for trimmed reads. (DOC) [file pgen.1002345.s025.doc]

**Table S20**. Sequencing statistics for trimmed reads.

|  | *P. lutzii* | Pb03 | Pb18 |
| --- | --- | --- | --- |
| 4kb (pOT) paired-end reads | 235,235 | 265,682 | 299,544 |
| 10kb (pJAN) paired-end reads | 77,886 | 93,671 | 4,627 |
| 40kb (Fosmid) paired-end reads | 37,493 | 37,868 | 107,498 |
| All libraries, paired-end reads | 350,614 | 397,221 | 411,669 |
|  |  |  |  |
| 4kb (pOT) total bases (Mb) | 180.7 | 184.3 | 223.9 |
| 10kb (pJAN) total bases (Mb) | 60.6 | 57.9 | 3.3 |
| 40kb (Fosmid) total reads (Mb) | 27.3 | 27.1 | 74.2 |
| All libraries, total bases (Mb) | 268.5 | 269.3 | 301.4 |
