# Supplementary material for: Comparative Genomic Analysis of Human Fungal Pathogens Causing Paracoccidioidomycosis
Source: PLoS Genet. 2011 Oct 27;7(10):e1002345. doi: 10.1371/journal.pgen.1002345 (PMC3203195; doi:10.1371/journal.pgen.1002345)
Supplement: Text S1 — Supplementary methods and text. (DOC) [file pgen.1002345.s026.doc]

**Text S1. Supplementary methods and text for “Comparative Genomic Analysis of Human Fungal pathogens causing Paracoccidioidomycosis”.**

**Supplementary methods and text:**

S1. Sequencing, assembly, and subtelomere location.

S2. Annotation.

S3. Single nucleotide polymorphism (SNP) identification.

S4. Mating and meiosis.

S5. Sterol biosynthesis.

S6. Identification of potential drug targets.

**S1. Sequencing, assembly, and subtelomere location:**

Three whole genome shotgun sequencing libraries were constructed from genomic DNA for each strain; two plasmid libraries (4 kb and 10 kb inserts) and a Fosmid library (40 kb inserts). Paired-end reads were generated for each using ABI/Sanger technology (Table S19). Assemblies were generated using Arachne [1], and mitochondrial sequence was separated out. The mitochondrial assembly of *P. brasiliensis* Pb03 includes all expected genes and is nearly complete, however the assembly of *P. lutzii* only covers 31 kb in sequence (Table S4).

To identify telomeric sequence, we searched for arrays at least 7 copies of the telomeric repeat (TTAGGG). For *P. brasiliensis* Pb18, the repeat is found at the start of scaffold 3, the end of scaffold 5, the start of scaffold 9, the end of scaffold 10, and the end of scaffold 16. These locations map to the ends of linkage groups in the optical map, supporting that these are telomeres. In addition, scaffold 27, which is not anchored to the optical map, contains a telomeric repeat array. In the other genomes, telomeric repeat is found at the end of scaffold 13, the start of scaffold 17, and the start of scaffold 100 in *P. lutzii*, and at the end of scaffolds 17, the start of scaffold 21, and the start of scaffold 24 in *P. brasiliensis* Pb03. To identify additional locations where small gaps separate the telomeric array from scaffold ends, we identified telomeric repeat arrays in the unassembled sequence, where the other read from the same clone was anchored in the assembly and the unassembled read was placed off the end of a scaffold. This linked telomeric sequence to the end of *P. brasiliensis* Pb18 scaffold 12 and the start of scaffold 13, both of which correspond to ends of linkage groups in the optical map. Also, telomeric repeat is linked to the ends of unlinked scaffolds 20, 21, and 26, suggesting they all correspond to subtelomeric regions. In combination with the telomeric repeat arrays found at the ends of *P. brasiliensis* Pb18 scaffolds, this supports the identification of 7 out of 10 telomeres in the assembly. In *P. lutzii*, telomeric repeat is linked to the start of scaffold 1, the start of scaffold 15, the end of scaffold 29, and the start of scaffold 66. In *P. brasiliensis* Pb03, telomeric repeat is linked to the start of scaffold 1, the start of scaffold 8, end of scaffold 12, start of scaffold 21, start of scaffold 24, end of scaffold 29, start of scaffolds 32, 33, 35, and 57.

**S2. Annotation:**

Protein coding genes in the *P. brasiliensis* and *P. lutzii* genomes were annotated by a combination of EST-based transcript identification, computational gene prediction programs, and manual revision of flagged calls. Over 49,000 ESTs available from GenBank or collaborators were aligned to each genome using BLAT, and the alignments were used to construct transcripts using in-house scripts. We also predicted potential genes using FgenesH [2], GeneID [3], GeneMark [4], GeneWise [5], and Augustus [6], which were trained using high confidence EST gene sets. The gene model with the best agreement with BLAST and EST evidence at each locus was selected. Gene models with potential problems were reviewed and edited if necessary, and manually edited gene models from collaborators were incorporated. Genes that did not fall into an orthologous group with any gene in the 15 taxon fungal data set (see gene family analysis), without a BLASTP hit of 1e-10 or lower against the GenBank non-redundant protein database, without a HMMER PFAM domain match of 1e-10 or lower, and without representative ESTs were flagged as likely false-positives. The initial gene set was filtered to remove transposable elements, identified via a combination of RepeatScout [7], RepeatMasker [8], Cross_match alignment of the genome against itself, transposon PFAM domains, transposable element gene product names, and BLAST similarity to an in-house database of proteins from transposable elements. Preliminary transcripts were also aligned to the genome assembly using BLAT with a 90% identity cutoff, and unnamed transcripts which aligned to five or more different loci were flagged as probable transposons and removed. Genes were named based on BLAST homology with greater than 30% query coverage and sequence identity.

Mitochondrial genomes for each strain were separated from the nuclear assembly and annotated separately. The *P. brasiliensis* Pb18 mitochondrial assembly [9] was used as a reference to identify all mitochondrial sequence in the other two strains. The *P. brasiliensis* Pb03 mitochondrial assembly totals 74 kb in size and contains a complete set of 16 predicted protein coding genes, including seven NADH dehydrogenase subunits (1, 2, 3, 4, 4L, 5, and 6) three ATP synthase subunits (6, 8, and 9), three cytochrome oxidase subunits (1, 2, and 3), and apocytochrome b, ribosomal protein S5, and a conserved maturase protein. The *P. lutzii* mitochondrial assembly totals only 31 kb in size; no shotgun reads were sequenced which cover the *NADH2*-*apoB*-*NADH3* region.

**S3. Single nucleotide polymorphism (SNP) identification:**

SNPs were identified between the *Paracoccidioides* strains by comparing the sequencing reads from the *P. brasiliensis* Pb03 and *P. lutzii* genomes with the *P.* *brasiliensis* Pb18 assembly. *P. brasiliensis* Pb03 and *P. lutzii* sequencing reads were aligned with the *P. brasiliensis* Pb18 assembly using Blat. Reads that did not have a unique placement in the assembly or had >20% gaps in the alignment were rejected. The Neighborhood Quality Standard (NQS) algorithm was used to identify high confidence polymorphic sites. SNPs were identified as single-base sequence variants that had a minimum PHRED base quality of 25 at the position of mismatch, a neighborhood base quality of 20, and no mismatches within 5 bp. The overall rate of SNPs for each genome was computed using the total aligned NQS positions as the denominator. For *P. lutzii* reads, the total number of aligned bases which satisfied the NQS criteria was roughly half that with the *P. brasiliensis* Pb03 reads, likely reflecting higher overall divergence than estimated by SNPs as more reads will not align across species. In no case were 3-alleles were called for any position. SNPs were submitted to dbSNP at NCBI and released in build id 133, with rs# 154774690-155410572. This data can be accessed at <http://www.ncbi.nlm.nih.gov/SNP/snp_viewTable.cgi?handle=BROAD-GENOMEBIO>. All SNPs have been released on the Broad *Paracoccidioides* website (http://www.broadinstitute.org/annotation/genome/paracoccidioides_brasiliensis/MultiHome.html).

To estimate the background level of false positive SNP calls, *P.* *brasiliensis* Pb18 sequencing reads were compared to the *P.* *brasiliensis* Pb18 assembly. Since these genomes are haploid, no SNPs are expected, so the number called is an estimate of the false positive rate. Our method identified a total of 385 positions as within Pb18 SNP variants; for this comparison, 95% of the assembly (27.9 Mb) contained aligned NQS bases. From this we estimate the false positive rate of SNP calling to be 1 SNP every 73 kb. By comparison the SNP rate between the two *P.* *brasiliensis* strains was estimated at 1 every 132 bases (see main text).

**S4. Mating and meiosis:**

We searched for orthologs involved in mating and meiosis in *S. cerevisiae*, *S. pombe*, *N. crassa*, and *A. nidulans* in the three *Paracoccidioides* genomes, *H. capsulatum* (Nam1), *C. immitis* (RS), and *A. fumigatus*. Potential orthologs were identified as either best BLAST hits, or best bi-directional BLAST hits between the two genomes. No genes were found to be missing only in the *Paracoccidioides* genomes. Some genes were missing in all the dimorphic fungi but found in *A. fumigatus*, including the ascus development gene *ASD1* from *N. crassa*, the helicase *SRS2* from *S. cerevisiae*, a predicted acetylglucosaminlytransferase (*SPBC4C3.08*) from *S. pombe*, and two meiosis specific transcription factors (*NDT80* and *UME6*). Additional genes were missing in a subset of species or in all the dimorphic fungi and *A. fumigatus* (Tables S12 and S13). The *Paracoccidioides* genomes all contain an ortholog of the karyogamy gene *KAR5* from *S. cerevisiae*, which is not present in the other dimorphs or in *A. fumigatus*.

**S5. Sterol biosynthesis:**

An analysis of several key genes whose products are involved in lipid synthesis revealed that they were present in the *P. brasiliensis* and *P. lutzii* genomes (Table S17). One of the major bottlenecks in ergosterol biosynthesis is the reaction catalyzed by HMG-CoA reductase, encoded by *HMG1* [10]. This gene and its deduced product showed slight differences in the *P. lutzii* isolate, a deduced protein longer (1,158 aa) than the corresponding proteins in the other two genomes (1,143 aa-1,144 aa). Squalene epoxidase and cytochrome P450-dependent C14 demethylase, encoded by *ERG1* and *ERG11*, respectively, are the main targets of allylamines (the former) and azoles (the latter), the most widely used antifungals. The isolate *P. lutzii* *ERG1* differs from the other two in number of introns in the gene and the length of its deduced protein (1,434 aa (*P. lutzii*) vs 1,596 aa (Pb03) or 1,566 aa (Pb18)), a difference that is also observed in *P. lutzii* *ERG11* (603 aa (*P. lutzii*) vs 526 aa (Pb03) or 524 (Pb18)). The C-5 sterol desaturase, encoded by *ERG3*, is nearly identical in all three genomes. *SMT* and *SMR* have similar nucleotide sequences and intron numbers and positions in all three genomes, as well as protein lengths (378 and 542-543 amino acids for Smt and Smr, respectively) (Table S17). By contrast, the enzyme delta 7-reductase, responsible for the modification of ergosterol into brassicasterol, is substantially different in all three genomes, as the number of introns varies from 3 to 5 in these orthologs, and the predicted amino acid length ranges from 427 to 517 (Table S17). This enzyme is of particular interest in *P. brasiliensis* because, in contrast to other fungal membranes, *P. brasiliensis* membranes have both brassicasterol and ergosterol as final products, the former being prevalent in the yeastlike phase and the latter in the mycelial phase [11,12].

**S6. Identification of potential drug targets:**

Potential drug targets were identified as genes conserved in the *Paracoccidioides* genomes but not in the human genome. These genes were manually curated to select a candidate list of drug targets (Table S18). Individual target rationale is described below.

Cell wall: glucans and chitin. Alpha-glucans are present in medically important human pathogenic fungi such as *Histoplasma* *capsulatum*, *P. brasiliensis* and *Blastomyces dermatitidis*. In these species, mutants with a reduction of α-(1,3)-glucan in their cell wall are avirulent [13,14,15]. In another human pathogen, *Cryptococcus neoformans*, inactivation of a unique α-glucan synthase gene leads to a significant decrease in the α-(1,3)-glucan content of the cell wall. *Cryptococcus* requires cell wall α-glucan to anchor its capsule, which is critical for virulence [16]. Despite their potential relevance for virulence, little is known about the biosynthesis of α-glucans in fungi. In *S. pombe*, the Mok11 α-(1,3)-glucan synthase is induced during the sporulation process, however deletion did not show a sporulation defect [17]. Those considerations support the interest in developing drugs to members of the glucan metabolism in fungi.

Cell wall: mannoproteins. The addition of *N*-linked and/or *O*-linked oligosaccharides is a frequent modification of cell wall proteins. Mannosyltransferases play a crucial role in this process and likely generate other glycoconjugates. The ScKre2/ScMnt1 α-1,2-mannosyltransferase of *S. cerevisiae* and the homologous enzymes CaMnt1 and CaMnt2 of *C. albicans* have been shown to be required for the addition of the second and third mannosyl residue of *O*-linked carbohydrates [18], and they play a pivotal role in the elaboration of the outer chains of *N*-linked glycans [19]. Highly glycosylated mannoproteins are involved in host cell adhesion, antigenicity, and modulation of host immune responses [20,21,22]. Studies showed that mannoproteins of the outer layer mediate direct interactions of *C. albicans* with host cells [23] and play important roles in pathogenesis [24]. These considerations make mannosyltransferases promising targets for novel antifungal therapies.

Cell membrane: ergosterol. Ergosterol is a component of fungal cell membranes, and is not present in animal cells. Established antifungal drugs target ergosterol or the biosynthetic pathway; azole drugs such as fluconazole target *ERG11* (lanosterol 14-alpha-demethylase) [25]. *ERG25* and *ERG28* were identified by this bioinformatic screen, and may represent additional targets.

Metabolic pathways: A diverse set of enzymes may represent good targets, particularly those not found in animal cells. Targets conserved in *Paracoccidioides* sp include:

- Aspartate β-semialdehyde dehydrogenase (ASADH) is an essential enzyme found in bacteria, fungi and higher plants. This NADP-dependent enzyme lies at the first branch point in the biosynthetic pathway through which bacteria, fungi and higher plants synthesize amino acids, including lysine and methionine from aspartate. Blocks in this biosynthetic pathway, which is absent in mammals, are lethal in bacteria [26]. Inhibitors of ASADH may therefore serve as useful antibacterial, fungicidal and herbicidal agents.
- The shikimate pathway is present in algae, higher plants, fungi, bacteria, but is absent in mammals [27,28]. Studies have suggested that the amino acid availability is limited in the vacuoles of macrophages. Accordingly, the aromatic amino acid biosynthesis pathway is an attractive target for the development of new drugs. Prephenate dehydratase catalyses the decarboxylation and dehydration of prephenate to form phenylpyruvate, which in turn is converted to phenylalanine.
- Lumazine synthase is an enzyme involved in riboflavin biosynthesis in many plants and microorganisms; the riboflavin biosynthesis pathway is not present in humans. The structure of lumazine synthase from *C. albicans* has been determined allowing modeling of inhibitor binding [29].
- Members of the thioesterase family participate in metabolic pathways, such as degradation of fatty acids and siderophore synthesis pathways [30,31]. Due to the low amount of free iron in the host tissues this class of enzymes may be useful drug targets.
- Compounds affecting nucleotide metabolism are potential drug targets. For organisms which are deficient in purine synthesis, the purine salvage pathway may be a specific target. Adenosine deaminase (ADA) is an essential enzyme in the purine salvage pathway, catalyzing the deamination of adenosine to inosine, and subsequently converted into purine nucleotides. Studies have demonstrated a conformational change in the structure of the *Plasmodium* ADA in comparison of mammalian apo and substrate complex structures, supporting structure-based drug design efforts [32]. In the *de novo* pathway of purine nucleotide synthase, GMP-synthase catalyzes synthesis of guanine nucleotides; inhibitors had been validated in *C. albicans* and *A. fumigatus* [33].
- Dihydrofolate reductase (DHFR) plays an essential role in the synthesis of purines and some amino acids. Inhibitors of DHFR are antiprotozoal and antibacterial therapeutics [34,35]. In fungi, inhibitors of DHFR in *Candida glabrata* have recently been described [36].
- Metabolism during the yeast parasitic phase of *P. brasiliensis* is preferentially directed to alcoholic fermentation [37], especially during the infectious process as demonstrated by transcriptional analysis [38]. Enzymes such as alcoholic dehydrogenase (ADH) could be a target for new anti-fungal drugs, and compounds targeting ADH had been developed for *Entamoeba hystolytica* [39].
- Inhibition F1-FO-ATPase by a benzodiazepine (BZ-423), blocks respiratory chain function and generates superoxide. In animal cells, this compound inhibits growth by proteasomal degradation of c-myc [40].
- Co-catalytic metallopeptidases such as the AAP aminopeptidase may provide good targets due their central role in several disease states, including bacterial infections [41].
- The disruption of the degradative protein process by the 20S proteasomal catalytic β-subunits has implications in a number of human diseases and has exposed the proteasome as an important therapeutic target [42].

Other potential targets are listed in Table S18, and a subset described here:

- Cytochrome P450s are involved in detoxification in fungi and have been described as a target for novel therapeutic compounds [43].
- Carbonic anhydrases are critical for the virulence of pathogens such as *Plasmodium falciparum*, *Mycobacterium tuberculosis* and *Cryptococcus neoformans* [44,45,46].
- Tyrosine phosphatases constitute a large class of enzymes, which are crucial modulators of tyrosine phosphorylation-dependent cellular events and malfunction in the enzyme activity is associated with many human diseases [47]. Studies suggested that inhibitors of tyrosine phosphatases represent an effective strategy to combat metabolic syndromes [48].
- Orthologs of the macrophage migration inhibitory factor have been implicated as playing roles in a number of diseases [49]. Proteins with a macrophage migration inhibitory factor PFAM domain are conserved in *P.* *brasiliensis* and *H. capsulatum*.
- Phospholipase D is required for virulence of *C. albicans* and *Corynebacterium pseudotuberculosis* [50,51].

**References for supplementary information.**
